# T lymphocyte cell: A pivotal player in lung cancer

**DOI:** 10.3389/fimmu.2023.1102778

**Published:** 2023-01-27

**Authors:** Yanan Wu, Meng Yuan, Chenlin Wang, Yanfei Chen, Yan Zhang, Jiandong Zhang

**Affiliations:** ^1^ Department of Oncology, the First Affiliated Hospital of Shandong First Medical University & Shandong Provincial Qianfoshan Hospital, Shandong Key Laboratory of Rheumatic Disease and Translational Medicine, Shandong Lung Cancer Institute, Jinan, China; ^2^ Department of Oncology, Shandong First Medical University & Shandong Academy of Medical Sciences, Jinan, China; ^3^ School of Clinical Medicine, Weifang Medical University, Weifang, China; ^4^ Medical Integration and Practice Center, Cheeloo College of Medicine, Shandong University, Jinan, China

**Keywords:** lung cancer, T lymphocyte, immunotherapy, tumor microenvironment, immunosuppression

## Abstract

Lung cancer is responsible for the leading cause of cancer-related death worldwide, which lacks effective therapies. In recent years, accumulating evidence on the understanding of the antitumor activity of the immune system has demonstrated that immunotherapy is one of the powerful alternatives in lung cancer therapy. T cells are the core of cellular immunotherapy, which are critical for tumorigenesis and the treatment of lung cancer. Based on the different expressions of surface molecules and functional points, T cells can be subdivided into regulatory T cells, T helper cells, cytotoxic T lymphocytes, and other unconventional T cells, including γδ T cells, nature killer T cells and mucosal-associated invariant T cells. Advances in our understanding of T cells’ functional mechanism will lead to a number of clinical trials on the discovery and development of new treatment strategies. Thus, we summarize the biological functions and regulations of T cells on tumorigenesis, progression, metastasis, and prognosis in lung cancer. Furthermore, we discuss the current advancements of technologies and potentials of T-cell-oriented therapeutic targets for lung cancer.

## 1 Introduction

**Figure 1:**
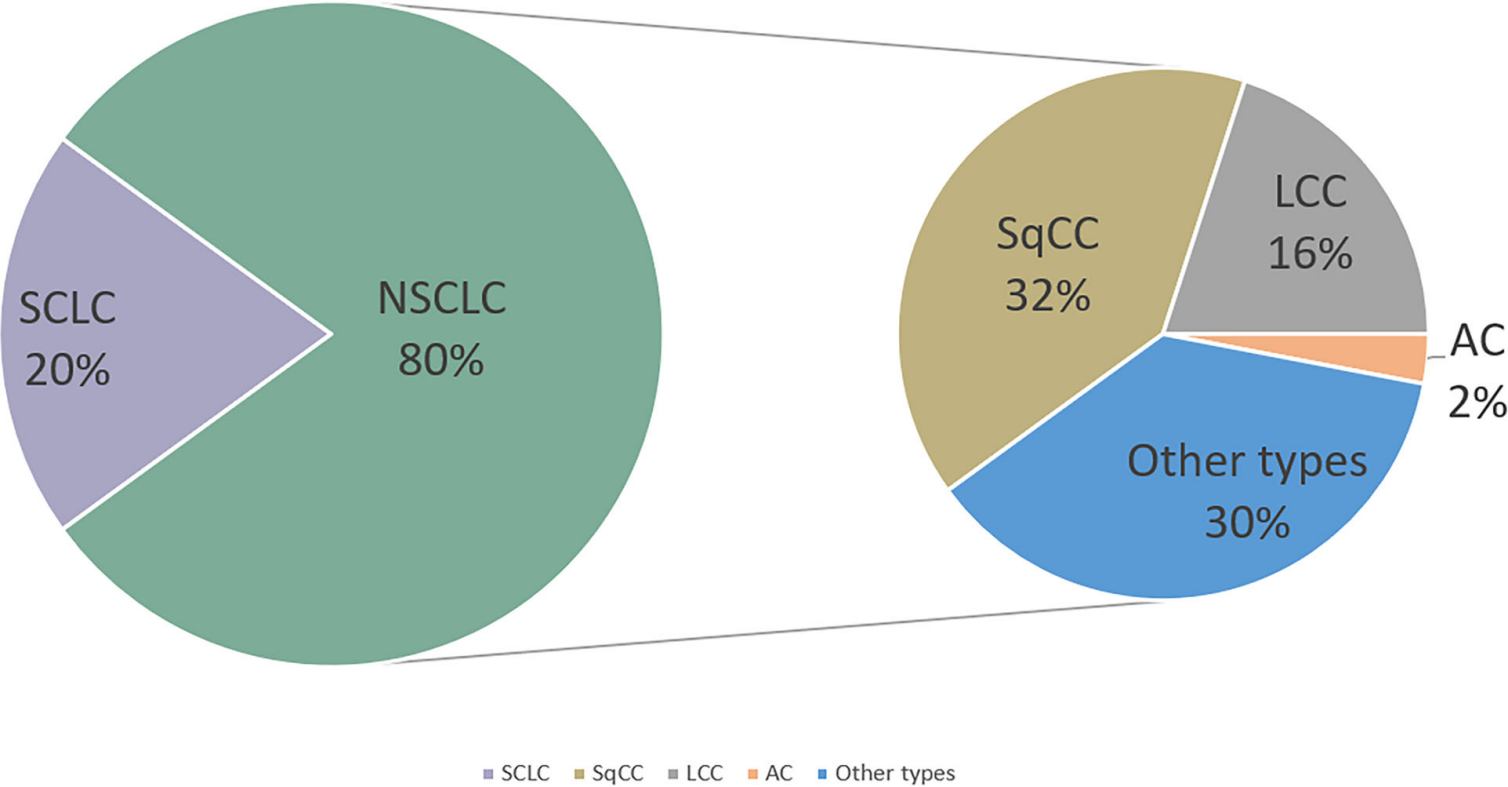
Lung cancer incidence with its classification. NSCLC, non-small-cell lung cancer; SCLC, small-cell lung cancer; SqCC,squamous cell cancer; LCC,large-cell carcinoma; AC, adenocarcinoma.

Lung cancer represents one of the most common malignancies and is the leading cause of cancer-related death worldwide (1). The 5-year survival rate (SR) of lung cancer patients is only 10%–20% (2). Lung cancer is classified into two subtypes: small-cell lung cancer (SCLC; accounts for 20%) and non-SCLC (NSCLC; accounts for 80%). Histologically, NSCLC can be primarily divided into adenocarcinoma (AC), squamous-cell carcinoma (SqCC), large-cell carcinoma (LCC), and other types (3) (**Figure 1**). Because of the lack of early typical symptoms and diagnosis indicators, majority of patients with lung cancer are often diagnosed with local invasion, lymph node, and distant metastasis at first-time consultancy. The traditional therapeutic strategy of lung cancer includes surgery, chemotherapy, and radiotherapy (4). Although the survival time of lung cancer patients can be extended with these therapies, the 5-year overall survival (OS) is still not satisfying (5). Thus, developing novel therapeutic strategies of lung cancer is urgently required. In recent years, as immune cells are crucial for both the development and treatment of cancer, people begin to understand cancer through the contribution of the immune system.

Quite a few studies have revealed that the development of lung cancer is closely related to immunological dysfunction. T cells are critical for tumorigenesis, which plays an important role in the treatment of lung cancer (6). In this context, investigating the number and function of T cells may be useful in developing novel therapeutic strategies about prolonging the survival time of patients. T-cell- associated cellular immunotherapy opens new possibilities for patients with lung cancer, bringing fresh treatment options for patients with advanced cancer or poor responses to traditional treatments.

**Figure 2:**
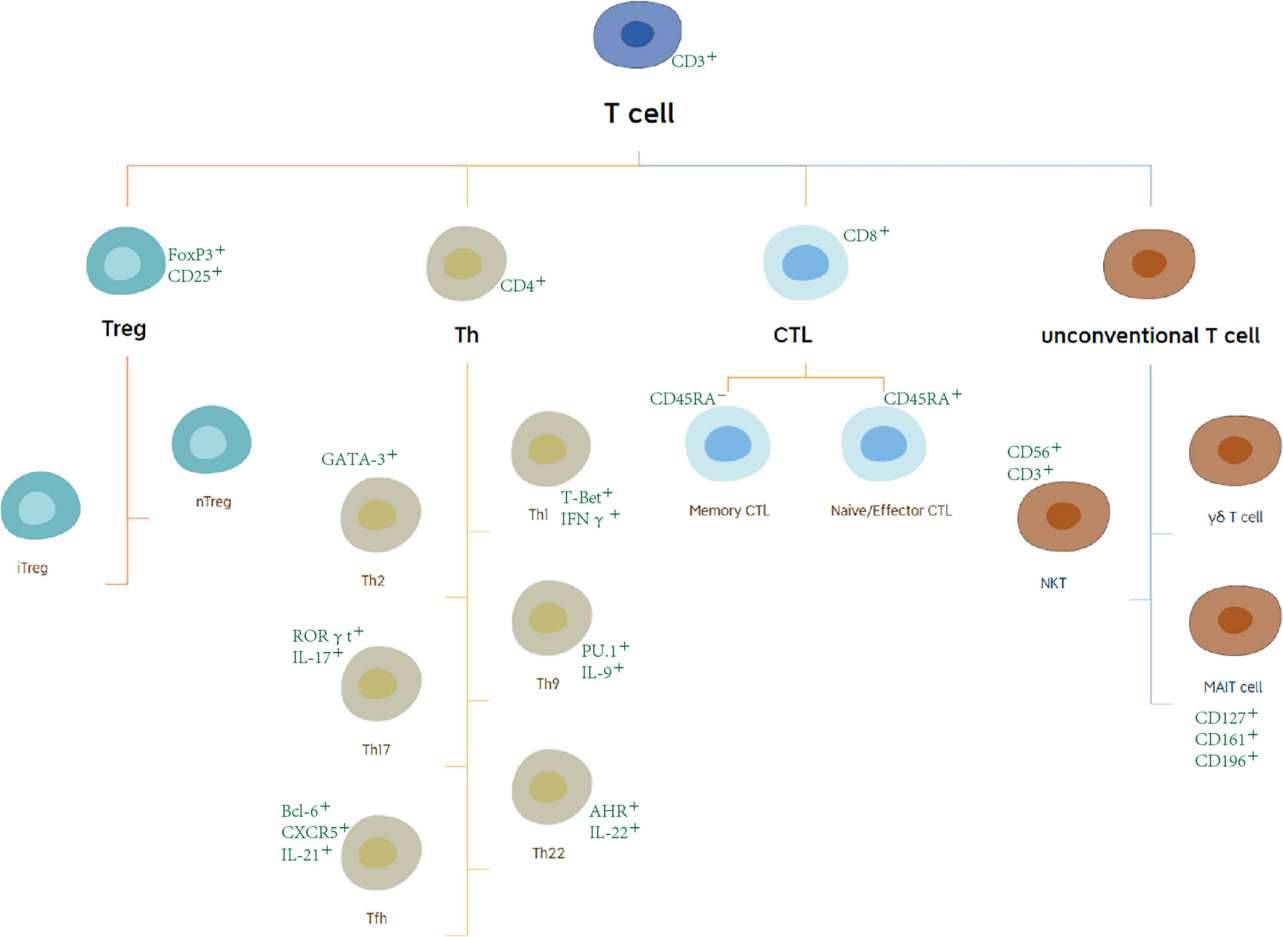
Subsets and surface marker of T cells. Based on the different expression of surface molecules and functional points, T cells can be subdivided into regulatory T (Treg) cells, T helper cells, cytotoxic T lymphocytes (CTLs), and other unconventional T cells.

T lymphocytes originate from bone marrow (BM) progenitors and then migrate to the thymus. After differentiating and maturing in the thymus, T lymphocytes distribute to the immune organs and tissues throughout the body to play an immune role through the lymphatic vessels, blood, and tissue fluid circulation (7). For the last few decades, T cells have been a research hotspot. At the present time, the characteristics of T-cell subsets have become clearer. Based on the different expression of surface molecules and functional points, T cells can be subdivided into regulatory T (Treg) cells, T helper (Th) cells, cytotoxic T lymphocytes (CTLs), and other unconventional T cells, including γδ T cells, nature killer T (NKT) cells, and mucosal-associated invariant T (MAIT) cells (8, 9) (**Figure 2**). Cell surface markers are special proteins that conveniently serve as the markers of specific cell types. We illustrate these in **Figure 2** (10, 11).

## 2 The role and mechanisms of T cells in lung cancer

### 2.1 Tregs and lung cancer

Tregs serve a significant function in the lung cancer progression and metastasis, which are considered as the biomarkers of poor treatment response in lung cancer patients. There have been several previous studies demonstrating that Tregs were accumulated or activated in lung cancer patients. In carcinogenesis, Tregs maintain immune tolerance to self-antigens, allow the tumor evasion, and contribute to disrupt antitumor immunity (12, 13).

Compared with healthy subjects, the percentage of Tregs in the tumor tissue, peripheral blood, and malignant pleural effusion is higher in patients with lung cancer. Patients with higher ratios of Tregs have a significantly worse survival (12, 14). Many lines of evidence suggest that Tregs promote the metastasis of lung cancer. Nasrollah Erfani et al. reported that the Treg level increased with staging, which was the highest in patients with metastatic tumors. Thus, Tregs can reflect the stage of lung cancer and predict the SR and recurrence (14, 15).

Tregs may suppress antitumor function by a number of different mechanisms. Previous studies have shown that Tregs can express soluble immunosuppressive cytokines or factors such as transforming growth factor-β (TGF-β), galectin-1 (Gal-1), IL-35, IL-10, prostaglandin E2 (PGE2), and adenosine or act through cell–cell contacts *via* high levels of cell surface molecules, including programmed cell death protein 1 (PD-1), programmed cell death ligand 1 (PD-L1), cytotoxic T-lymphocyte-associated antigen 4 (CTLA-4), lymphocyte-activation protein 3 (LAG-3), CD39/73, and neuropilin 1 (Nrp1), inducing immune dysfunction (16, 17) (**Table 1**). Mouse models revealed that Tregs partially repress the antitumor activity of CD8+ T cells. The depletion of Tregs in mice bearing lung AC (LUAD) treated with anti-CD25-depleting mAb restrained lung tumor growth and enhanced the recruitment of CD8+ T cells, leading to the upregulation of granzyme A, granzyme B, perforin, and tumor cell death (32). Furthermore, Tregs can suppress other effective immune responses. Murine models of Lewis lung carcinoma have demonstrated that Tregs directly suppressed the ability of NK cells to clear tumor cells by a TGF-β-dependent mechanism. Forkhead box protein P3 (Foxp3) is a recognized marker of Tregs that plays a significant role in maintaining the immunosuppressive function of Tregs (33). Foxp3 overexpression induces the viability and invasiveness of lung cancer cells, resulting in tumor cell growth. Peng et al. isolated Tregs from the peripheral blood of eight healthy volunteers and then cocultured them with the human NSCLC cell line 95 D. The results showed that matrix metalloproteinase-9 (MMP-9) expression in tumor cells was increased and apoptosis was decreased following coculture with Tregs. Researchers transfected Tregs with the plasmid-overexpressing Foxp3 in order to construct the Foxp3-overexpressing Tregs. The results demonstrated that the tumor invasive ability and the viability of 95D cells were significantly increased. In addition, the expression of Foxp3 in Tregs may be associated with the complex interaction between various immune cells and tumor cells. Tumor cells secrete a great number of cytokines that induce the sustained expression of Foxp3, resulting in immunosuppression. For example, JC et al. reported that TGF-β1 maintains the expression of Foxp3 and stabilizes the number and functions of Tregs (34). SCLC tumor cells are involved in Treg induction by the secretion of IL-15, which could promote Treg cells’ immunosuppressive functions (21). Intrinsic resistance and acquired resistance to chemotherapy in patients with lung cancer are one of the main causes of high mortality. The lung cancer microenvironment is enriched in Tregs, which may explain the chemotherapy resistance of lung tumor cells. Mesenchymal cells low on E-cadherin are more chemoresistant. The experiments indicated that there is a dynamic and inverse correlation between downregulation in the levels of CDH1 (encoding the epithelial cell marker E-cadherin) and the observed increase in the IL2RA (encoding the Treg marker CD25) in stage IV lung cancer patients resistant to chemotherapy (35). Moreover, the finding revealed Tregs to prompt Kras-related immunosuppressive chemoresistance associated with the CD8+ T-cell exhaustion phenotype (36).

**Table 1:** Summary of the cytokines associated with T cells in lung cancer.

Cytokines	Cell	Role in lung cancer	References
IL-1	Myeloid cell	Protumor	(18)
IL-2	Th1 cell	Antitumor	(19)
IL-9	Th9 cell, Treg	Protumor	(16, 17, 20)
IL-10	Treg	Protumor	(16, 17)
IL-15	Tumor cell	Protumor	(21)
IL-17	Th17 cell, γδT cell	Protumor	(22, 23)
IL-21	Th9 cell, Th17 cell, Tfh	Antitumor	(24–26)
IL-22	Th17 cell, Th22 cell	Protumor	(27)
IL-26	Th17	Protumor	(28)
IL-38	Tumor cell	Protumor	(29)
TNF-α	Th1 cell, CTL	Antitumor	(19, 30)
IFN-γ	Th1 cell, CTL	Antitumor	(19, 30)
TGF-β	Treg, tumor cell	Protumor	(16, 17, 31)
PGE2	Treg	Protumor	(16, 17)

In addition to Foxp3 Tregs, numerous newly identified subpopulations of Tregs are involved in suppressing antitumor immunity, for example, Forkhead box protein A1(FOXA1+). Tregs express elevated levels of FOXA1+ but low levels of FOXP3+. Although the detailed function of FOXA1+ in immunity is still not completely understood, previous *in vitro* suppression assays reported that FOXA1+ Tregs inhibited the antitumor immune responses of T cells and induced the expression of PD-L1. Considering the PD-L1 expression level severed as a biomarker for anti-PD-1/PD-L1 therapy response, FOXA1+ Tregs may provide clinical value in identifying potential targets for a new treatment strategy (37). C-C chemokine receptor 8 (CCR8) is a chemokine receptor that is selectively expressed on tumor-infiltrating Tregs in NSCLS. Increased CCR8+ Treg infiltration is associated with a poor clinical outcome in lung cancer. Compared with CCR8-Tregs, CCR8+ Tregs express higher molecules that are associated with immunosuppression function. CCR8+ Tregs enhance chemokines, leading to recruit effector T cells to surroundings and suppress their activation (38). CCR8+ Tregs may form a Treg accumulation site by highly expressing chemokine and its receptors, leading to the formation of an immunosuppressive environment (39). Moreover, Haruna found that the CCR8+ cell depletion from tumor-infiltrating cell (TIC) culture enhances the function of CD8 T cells (38).

Taken together, these studies verified the role of Tregs in the development of lung cancer and provide plenty of evidence in the relationship between lung metastatic burden and Tregs. Further studies are warranted to understand the immunosuppressive functions of Tregs.

### 2.2 T helper cells and lung cancer

Th cells are an important component of our immune system that help other immune cells to eliminate pathogens. Effector Th cells are determined by the type of cytokine released by the antigen-presenting cell (APC). The subtypes of effector Th cells include Th1,Th2, and Th17. Recently, some literatures have discovered other populations such as Th9, Th22, and T follicular helper (Tfh) cells (40).

The Th1 cell is the first subset discovered in CD4+ T cells. Th1 cells, which release tumor necrosis factor alpha(TNF-α), IL-2, and interferon gamma (IFN-γ), contribute to antitumor immune responses in lung cancer (19). Th1 cells play an important role in the activation of CD8+ T cells and NK cells. IFN-γhas an antitumor effect while promoting apoptosis through positively regulating the activation state of STAT1 (41, 42). However, Ito et al. detected the irrelevance between Th1 and IFN-γ production in tumor-infiltrating lymphocytes (TILs), supporting the claim that the function of tumor-infiltrating Th1 cells is suppressed in lung cancer (42, 43). Apart from Th1 cells, other subpopulations, in particular Th9 cells (producing IL-9 and IL-21) and Th17 cells (producing IL-17), have been implicated in lung cancer. Compared with non-tumor samples, these two types of cells are enriched in the tumor tissue and malignant pleural effusion (MPE) of patients with lung cancer (41, 44). Th9 cells were originally identified as CD4+ T-cell subsets in 2008 (45). IL-9 is highly expressed in NSCLC and plays a key protumoral role in NSCLC (20). Salazar et al. reported that Th17/IL-17 and Th9/IL9 alters the gene expression profile in lung cancer cells to induce epithelial mesenchymal transition (EMT)and promote metastasis and angiogenesis tumor migration. It has been revealed that, by activating STAT3 signaling, IL-9 strongly induced tumor cell proliferation and migration, prevented tumor cells from apoptosis induced by IFN-γ, and facilitated the intercellular adhesion of tumor cells to pleural mesothelial cell monolayers (PMCs) *via* ICAM-1/LFA-1- and/or VCAM-1/integrin-β-dependent mechanisms. In addition to IL-9, Th9 cells produce another cytokine, IL-21, which has antitumor effects. It has been reported that CD4+ Th cells producing IL-21 supported the effector functions of CD8+ TIL *via* IL-21/IL-21R signaling (24). A study demonstrated that serum IL-17 levels were increased in the NSCLC group compared to the healthy control group (46). Chen et al. noted that the enhanced appearance of IL-17 producing cells was significantly correlated with poor patient survival (47). IL-17 levels are much higher in lung cancer MPE than heart failure pleural effusions, which are negatively associated with the survival levels of lung cancer patients (48). In NSCLC, IL-17 has a direct effect on the metastasis of cancer cells both *in vitro* and *in vivo* (22). In addition to IL-17, Th17 cells produce other proinflammatory cytokines such as IL-21, IL-22, and IL-26, which are also related with lung cancer (25, 28, 49). IL-22 is highly expressed in tumor tissue, blood, and malignant pleural effusion in NSCLC. Studies revealed that the overexpression of IL-22 induced lung cancer cell resistance against apoptosis *via* the activation of STAT3 and the inactivation of ERK ½. IL-22 is not only secreted by Th17 cells but also involved in Th22 cell-mediated immune response (27). IL-26 is one of the latest discovered IL-10 family members mainly secreted by Th17 cells. The recently study reported that IL-26 is involved in the production and promotion of MPE by enhancing CD4+IL-22+ T-cell differentiation and inhibiting CD8+ T-cell tumor-killing activity (28). However, several lines of evidence suggest that Th17 cells may also paradoxically contribute to the control of the immune response. IL-21, which is produced by Th17 cells, can expand CD8+ T cells, leading to tumor regression in NSCLC and melanoma (25) Moreover, Th17 cells are found to promote protective antitumor immune responses by promoting effector T-cell, dendritic-cell (DC), and NK-cell tumor microenvironment (TME) recruitment and retention within the TME (50). Studies have shown that high counts of Th17 cells in MPE predicted increased patient survival in NSCLC (51).

At present, cancer immunology has shifted from individual cell populations to the integrated understanding of potential interactions among immune cell populations. Th17 cells and Treg cells share a common precursor cell (the naïve CD4+ Th precursor cell), requiring the common signaling pathway mediated by TGF-β for initial differentiation (52). Th17 and Treg cells are considered to inhibit autoimmunity. Thus, the reciprocal generation of these two CD4+ cells is critically important. Studies have shown that the balance of Th17/Treg cells plays a critical role in the development, progression and prognosis of lung cancer. Similar to Tregs, the percentage of Th17 is significantly higher in the peripheral blood and MPE of patients with NSCLC than those in the healthy control group (41, 53). However, the balance of T17 cells and Treg cells is broken in NSCLC. Th17/Treg frequency was found to be much greater in patients with NSCLC compared to those in healthy controls, with an inversely correlation between these two CD4+ cells. It has been proven that the high ratio of Tregs to Th17 is significantly predictive of the poor prognosis of lung cancer patients (54). The external milieu (e.g., cytokines) presented during activation determine the final fate of T-cell subsets. In the NSCLC microenvironment, IL-1β, IL-6, and IL-23 are critical in maintaining and expanding Th17, while TGF-β1 and IL-10 are known to positively correlate with Tregs. However, TGF-β1 is the inhibitor of Th17 differentiation (53). Given the plenty of evidence above all, the balance of Th17/Treg cells is broken with the tumor progression in lung cancer, which indicates that further studies are required to understand the mechanism underlying the function and balance of Th17 and Treg cells in NSCLC. Reversing the imbalance of Th17/Treg modulation may provide an effective strategy for lung cancer therapy.

The circulating T follicular helper (Tfh) cell is considered as a novel CD4+T cell subset. Tfh cells are specialized B-cell helpers during the humoral immune response, which help the B-cell activation of germinal center and antibody production (55). Moreover, Tfh cells produce IL-21, which increases antitumor immunity by promoting the expression of IFN-γ and granzyme B in tumor-infiltrating CD8+ T cells (26). In NSCLC patients, the frequency and function of Tfh cells in peripheral blood are significantly lower compared to healthy controls. Prognostically, the frequency of Tfh cells in the tumor tissue of NSCLC patients has been linked to longer survival time from the date of surgery. The results suggested that the Tfh cells were likely involved in antitumor immunity through a potentially specific pathway and associated with a favorable prognosis but suffered strong functional inhibition in NSCLC (56). In conclusion, restoring the cytokine network and Tfh balance may be a novel and effective tool for the treatment of lung cancer.

Collectively, these findings suggest that Th cells play an important role in lung cancer immune regulation. The interaction between Th cells and tumor cells will further promote the current understanding of lung cancer.

### 2.3 Cytotoxic T cells (cytotoxic T lymphocytes and CD8+ T cells) and lung cancer

Cytotoxic T lymphocytes (CTLs) are an important immune component for controlling and killing tumor cells (57). However, studies that examined the relationship between CD8+ TILs and the prognosis in NSCLC remain controversial, varying with the distribution site, pathological classification, or methods used for cell quantification. Several studies have shown that high levels of CD8+ cytotoxic T cells within the tumor and tumor stroma are associated with a favorable lung cancer prognosis (58, 59). In another study, CD8+ T cells were associated with a favorable prognosis, which was only observed in SqCCs (60). However, Bonanno et al. have pointed out that the value of CD8+ TITLs in predicting lung cancer prognosis is limited. There was no correlation between the rate of CD8+ TITLs and the survival status (61). Other studies argued that only the density of stromal CD8+ T cells was consistently and significantly associated with longer survival (62). In 2022, Rodas and coworkers provided direct evidence that, in patients with NSCLC, the CD8+ T cells in stroma were higher in PD-L1-positive cases than in PD-L1-negative cases. Furthermore, the rate of CD8+ T cells was positively related to longer progression-free survival (PFS) and OS in patients with PD-L1-positive NSCLC treated with immune checkpoint inhibitors (ICIs) (63). In addition, the set of features related to tissue architecture was found to be associated with the recurrence of lung cancer. Corredor et al. exploited a set of descriptors (SpaTIL) that capture the local density and colocalization of TIL and tumor cells *via* digital images, predicting the likelihood of recurrence in early-stage NSCLC (64). Considering the heterogeneity of intertumoral localization of CD8+ T-cell infiltrations in NSCLC, the validly of different tumor sampling strategies would differ substantially. Small biopsies may not reliably represent the whole tumor. Taking a random sampling of 10–20 small areas or large biopsies may be practical choices for studies (65).

In order to kill the cancer cells, CD8+ cytotoxic T cells exhibited reactivity against tumor antigens (TAs) (66). TA peptides are presented to the APC surface with the form of the peptide major histocompatibility complex(pMHC). The activated CTL can specifically recognize pMHC through T-cell receptors (TCRs) and then kill their targeted cells *via* the granule exocytosis pathway or the Fas ligand (FasL) pathway (57). This form of antigen recognition is critical because antigen loss on tumor cells is responsible for recurrence after remission. Neoantigens are abnormal proteins produced by genetic mutations in cancer cells. They are ideal targets for T cells to recognize tumor cells that stimulate a violent antitumor immune effect. In general, the greater number of gene mutations in tumor tissue (the higher TMB), the more neoantigens carried (67). CD8+ T cells functioning as CTLs are an important component part of the cellular immune response that contribute positively to antitumor immunity.

**Figure 3:**
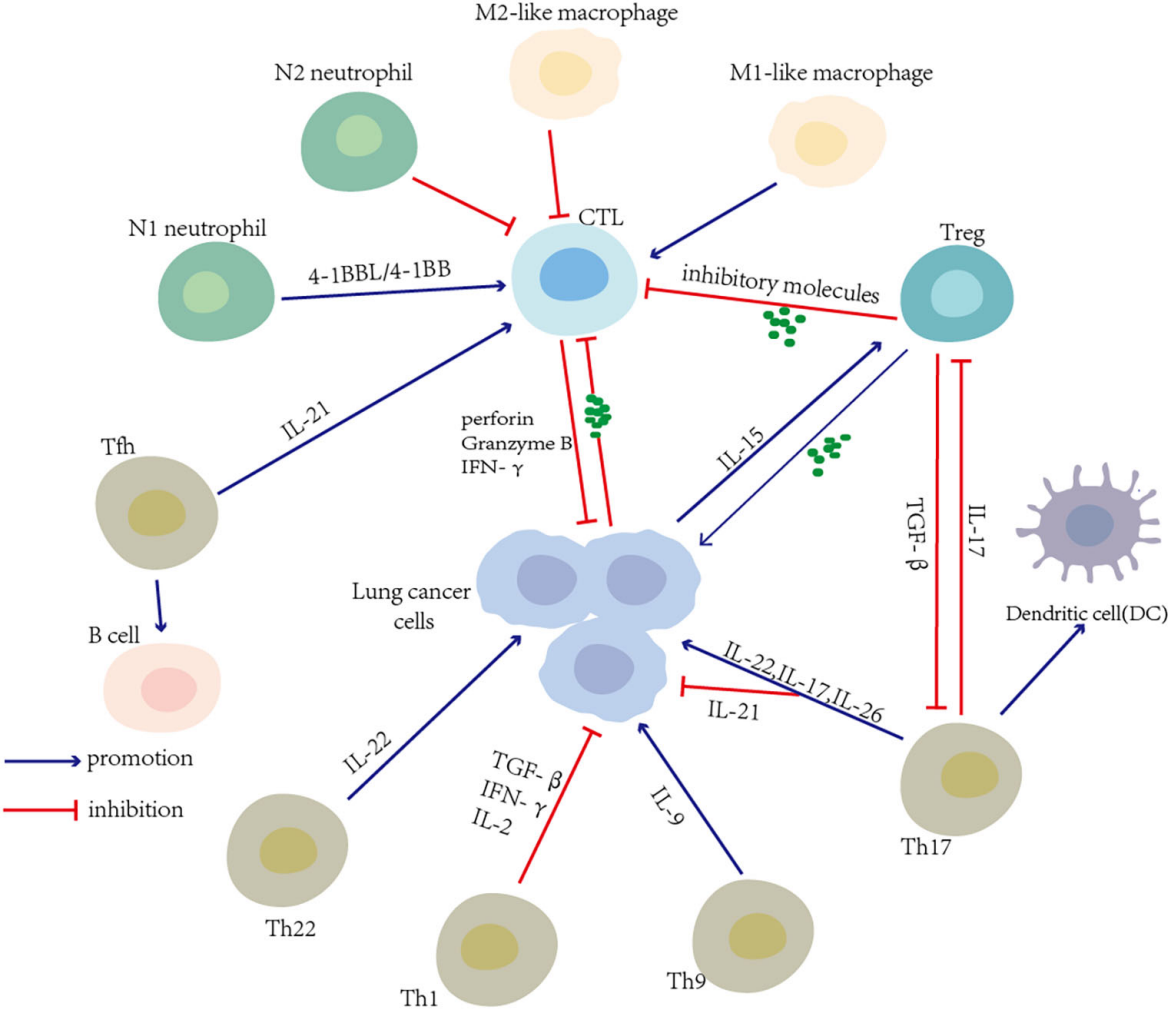
T-cell-associated interaction network in the lung cancer microenvironment. Various T-cell subsets and other immune cells in the tumor microenvironment (TME) influence each other and induce an antitumor or protumor immune response. Tumor cells themselves produce cytokines that favor the expansion of immune cells with suppression activity and induce cytotoxic T-cell dysfunction.

Ideally, the efficient activation of effector CD8+ T cells can lead to tumor cell death. However, previous research has reported that compared with healthy donors, the CD8+ T cells from lung cancer patients have a compromised capacity to proliferate and progressively lose the effector function, including reducing cytokine production capability (68). As noted by Xu, the levels of CD95, CD38, and perforin in circulating CD8+ T cells were decreased in lung cancer patients in stage III–IV. This result demonstrated that the function of activated CD8+ T cells was damaged in late-stage lung cancer. Lung cancer induces the alterations of CD8+ T cells. It is the impairment of antitumor immunity that results in a worse prognosis (69). Tumor cells release TAs, and soluble inhibitor factors may lead to a dysfunction of CD8+ T cells, resulting in immunological tolerance and hyporesponsiveness to host immunity. The recruitment of Tregs and other immune cells with suppressor activity by the lung tumor may induce the dysfunction of CD8+ T cells. The alternative immune checkpoints such as PD-1, CTLA-4, T-cell immunoglobulin domain and mucin domain-3 (TIM-3), B- and T-lymphocyte-associated (BTLA), which were detected on cytotoxic CD8+ T cells with a gradual and continuous upregulation, impair the recognition and killing of tumor cells (70). Moreover, in the malignant pleural effusions of lung cancer patients, the upregulation of the Fas ligand (FasL), TNF-related apoptosis-inducing ligand (TRAIL) expressions, and the low B-cell lymphoma-2 (Bcl-2) expression cells may be responsible for the activation-induced cell death that impairs the antitumor function of CD8+ T cells (68, 71). The more detailed mechanism of dysfunctional T cells can be found below. The restoration of CD8+ T-cell exhaustion or dysfunction is one of the focuses of therapeutic approaches for NSCLC (**Figure 3**).

### 2.4 T-cell dysfunction and lung cancer

**Figure 4:**
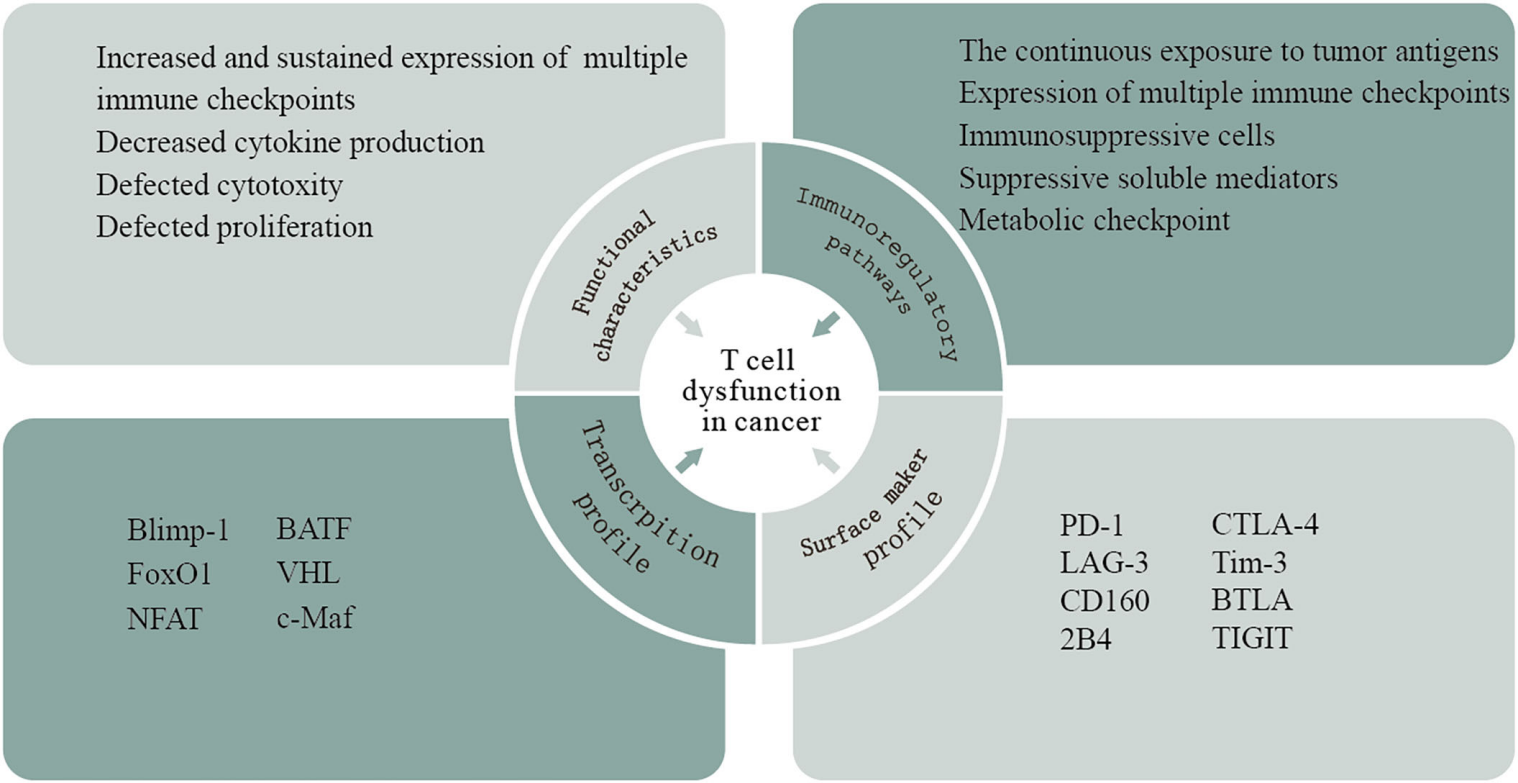
T-cell dysfunction in cancer. T-cell dysfunction has been described as a hyporesponsive response of various suppressive signals occurring in the TME. This acquisition of the dysfunctional state is driven by multifaceted immunoregulatory pathways in the TME. Based on the phenotypical, transcriptional, and functional analyses of T cells taken from patients with cancers, researchers have shown that these cells exhibit a particular gene expression profile that is different from those of naive, effector, or memory T cells.

T cells can recognize TAs expressed by cancer cells and play an essential role in tumor rejection. Although the presence of TILs, especially CD8^+^ T cells, is usually a marker of positive clinical outcomes in multiple solid tumors, the high frequency of these cells often fails to effectively promote tumor regression. In the context of high tumor-antigen load and immunosuppressive factor exposure in cancer, intratumoral T cells exhibit dysfunctional states (72). T-cell dysfunction has been described as a hyporesponsive response of various suppressive signals occurring in the TME, and, based on the phenotypic, functional, and molecular features of T cells, several other terms such as exhaustion, senescence, tolerance, and anergy are widely used to describe the dysfunctional state (73). Exhausted T cells (Texs) are the representative of T-cell dysfunction in the TME (74). The concept of T-cell exhaustion was originally identified in chronic lymphocytic choriomeningitis virus (LCMV) infection in murine models and was subsequently observed in human chronic viral infection and cancer (75).This acquisition of the dysfunctional state is driven by multifaceted immunoregulatory pathways in the TME, including (i) the continuous exposure to tumor antigens. Tumor-specific T cells are exposed to high antigen burden and consequent chronic antigen exposure, which may present sustained stimulation leading to repeated activation of T cells and ultimately T-cell dysfunction. (ii) The expression of multiple immune checkpoints. In general, T-cell function is impaired with increasing coexpression of inhibitory receptors. (iii) Immunosuppressive cells in the TME induce T-cell dysfunction, such as Tregs, myeloid‐derived suppressor cells (MDSCs), cancer-associated adipocytes, and fibroblasts. (iv) Suppressive soluble mediators. Some soluble molecules produced in the TME contribute to T-cell dysfunction such as IL-10, indoleamine-2,3 dioxygenase (IDO), TGF-β, adenosine, lactate, vascular endothelial growth factor A (VEGFA), and colony-stimulating factor (CSF). (vi) Physiological changes in the TME within hypoxia, low pH, and low nutrient levels (**Figure 4**).

Based on the phenotypical, transcriptional, and functional analyses of T cells taken from patients with cancers, researchers have shown that these cells exhibit a particular gene expression profile that is different from those of naive, effector, or memory T cells. The typical sign of T-cell dysfunction is the increased and sustained expression of multiple immune checkpoints, including PD-1, CTLA-4, LAG-3, Tim-3, CD160, BTLA, 2B4, and T-cell immunoreceptor with Ig and ITIM domains (TIGIT). Now, it is appreciated that the degree of coexpression of these inhibitory receptors directly correlates with the development of T-cell dysfunction. Accumulated evidence showed that interference with many of these receptors could reverse dysfunctional CD8+ T cells and improve tumor growth control *in vitro* and *in vivo (* 74). In addition, dysfunctional T cells undergo a hierarchical loss of effector functions, including early defects in IL-2 secretion, proliferation, and cytotoxicity, followed by the loss of β-chemokine, TNF-α, and IFN-γ production at later stages (76). The transcriptional regulation of T-cell dysfunction involves changes in the expression patterns and transcriptional connectivity of some key transcription factors. A number of transcription factors in chronic viral infection, including Eomes, Blimp-1, T-bet, BATF, FoxO1, VHL, NFAT, and c-Maf, are expressed in both functional and dysfunctional T cells. However, these transcription factors in dysfunctional T cells are linked to different genes and transcriptional loops, acting in a context-specific fashion (74). Notably, tumor-specific dysfunctional T cells display reduced Eomes and T-bet, which is different from those observed in chronic viral infections (77). Thymocyte selection-associated high mobility group box (TOX), a new transcription factor, revealed its crucial role in promoting T-cell dysfunction (78). Previous research demonstrated that the expression of TOX is high in dysfunctional tumor-specific CD8+ T cells, which is driven by nuclear factor of activated T-cell (NFAT) activation and chronic TCR stimulation (79). In tumor-specific TILs from mice and humans, TOX expression is positively correlated with the coexpression of inhibitory receptors and the low production of inflammatory cytokines (80). Several lines of evidence indicated that epigenetic imprinting plays an important role in T-cell dysfunction. Normally, encoding PD-1 (PDCD1) is impermanently demethylated in activated T cells; afterward, normal methylation levels are restored. However, it is reported that profound and persistent PDCD1 demethylation occurs in dysfunctional virus-specific T cells. Epigenetic therapy with the DNA hypomethylating agent azacytidine (AZA-Vidaza) induces the susceptibility of tumor cells to immune attack by T cells *via* upregulating genes and pathways related to immune evasion in several human cancer cell lines and genes related to both innate and adaptive immunity (81, 82). The combination of epigenetic therapy and PD-1 blockade has a synergistic antitumor response by enhancing T-cell responses and tumor control (83). In addition, the early dysfunctional TIL-encountering tumor antigen is differentiated to partial plastic in which T cells can be rescued by anti-PD-1 therapy and then transitioned to a fixed dysfunctional state that is resistant to reprogramming (84).

Targeted immunotherapies for treating tumor-induced T-cell dysfunction have provided clinical benefits to patients with cancer. At present, these immunotherapies have focused on immune checkpoint blockade using anti-PD-1 and/or CTLA-4 monoclonal antibodies, which are beneficial for patients with various solid and hematological tumors. With the improved understanding of the mechanisms leading to tumor-induced T-cell dysfunction, novel combination immunotherapies will emerge to reactivate dysfunctional T cells and improve the outcomes of cancer patients. However, there is considerable heterogeneity between patients and even within tumors. Thus, one major challenge is to identify the mechanisms of T-cell dysfunction for each patient to develop individualized immunotherapy in the future.

## 3 The regulation factor for T cells in lung cancer

T cells are the cores of tumorigenesis and cellular immunotherapy in lung cancer. However, many studies suggested that various factors regulated the function of T cells during lung cancer progression. We summarized the interaction of T cells and various effecting factors based on previous studies in lung cancer (**Table 2**).

**Table 2:** Summary of the regulation factors for T cells in lung cancer.

Factors	Role in lung cancer	Mechanism	References
IL-1	Protumor	Recruited γδT cells to secrete IL-17	(18)
IL-2	Antitumor	Reverse CD8+ T-cell exhaustion in MPE and shift from Th2 to Th1	(85, 86)
IL-12	Antitumor	Shift from Th2 to Th1	(85)
IL-15	Antitumor	Activate tumor-infiltrating bystander CD8+ T cells	(87)
IL-38	Protumor	Reduce the density of CD8+ TILs and the expression of intratumoral inflammatory cytokines	(29)
Surgical trauma	Protumor	Promote Treg recruitment *via* CCL2 expression	(88)
N1 neutrophil	Antitumor	Induce the proliferation and activation of CD4+ and CD8+ T cells in the early stage	(89)
N2 neutrophil	Protumor	Restrain effector CD8+ T-cell functions *via* CXCL-5	(89)
M1-like macrophage	Antitumor	Recruit the CD8+ T cells and NK cells by releasing chemokines and cytokines	(90)
M2-like macrophage	Protumor	Enhance differentiation toward Tregs and promote the infiltration and function of Tregs	(91)
MHC 1	Antitumor	Promote T-cell infiltration	(92)
MHC 2	Antitumor	Promote T-cell infiltration and/or T-cell retention in the TME	(93)
sCD100	Antitumor	Enhance CD8+ T-cell immune responses	(94)
Commensal Microbiota	Protumor	Induce the proliferation and activation of γδT cells	(23)
Curcumin	Antitumor	Suppress the Foxp3 activities in Tregs	(95)
Resveratrol	Antitumor	Reduce G-MDSC accumulation, enhance CD8+ T cells’ cytotoxic activity and Th1 immune responses *via* decreasing the expression of PD-1 on pulmonary T cells	(96, 97)

### 3.1 Cytokines

Cytokines produced in the TME regulate T-cell activation and infiltration. As shown in **Table 2**, IL-1 secreted by the cells of the myeloid lineage recruits γδ T cells to secrete IL-17 in the lungs of tumor-bearing mice without the involvement of Th17 cells, which may act to induce immune suppression (18). NSCLC cells are capable of restraining the proliferation and immune function of TILs by releasing a variety of immunosuppressive factors, such as transforming growth factor beta 1(TGF-β 1) (31). The activation of IL-2 and IL-12 could shift from Th2 to Th1, which reverses the immunosuppressed state (85). One of the accompanying manifestations of advanced lung cancer is MPE. MPE is correlated with a poor prognosis, whereas MPE CD8+ T cells are in exhausted conditions. Recently study identified that IL-2 can reverse CD8+ T cells with the exhaustion phenotype in the MPE of lung cancer (86). CD8+ TILs include both tumor antigen-specific CD8+ T cells and bystander CD8+ T cells. IL-15 stimulates bystander CD8+ T cells releasing IFN-γ in an NKG2D-dependent manner in NSCLC (87). Furthermore, the overexpression of IL-38 in tumor cells strongly promotes tumor growth through reducing the density of CD8+ TILs and the expression of intratumoral inflammatory cytokines in LUAD patients (29). More attention should be paid to the cytokines of lung cancer in the future.

### 3.2 Surgical trauma

Even though the surgical removal of tumors is a traditional treatment for lung cancer, both animal and clinical trials have suggested that surgical trauma has impact on promoting tumor progression (98, 99). So far, the mechanism has not been well elucidated. The key problem in tumor progression is the existence of an immunosuppressive microenvironment that helps tumor cells escape immune surveillance, thereby leading to tumor progression and metastasis. Postoperative inflammatory response can lead to a similar immunosuppressive state (100–102). Zhao et al. have shown that the surgical trauma contributes to promote excessive Treg recruitment by upregulating C-C motif chemokine ligand 2 (CCL2) expression. They indicated that CCL2 markedly promoted the percentage and migration of Tregs, whereas anti-CCL2 antibodies significantly inhibited the percentage and migration of Tregs (88). Nevertheless, far-too-little attention has been paid on the related signaling pathway and the molecular mechanism by which CCL2 promotes Treg recruitment. To sum up everything, surgical trauma leads to the lung cancer progression and metastases, and this finding provides knowledge in understanding the progression of lung cancer and identifying potential targets for a new treatment strategy.

### 3.3 Tumor-associated neutrophils

Tumor-associated neutrophils (TANs) account for a large proportion of the inflammatory cell population in lung cancer. In mouse tumor tissue, a number of studies have suggested that TANs may have dichotomous types of antitumor “N1 neutrophils” and protumoral “N2 neutrophils” (103). In 2014, researchers have suggested that the majority of TANs recruited in the TME in early-stage lung cancer patients are capable to stimulate CD8+ T-cell proliferation and responses. TANs express an antitumor (N1-like) activated phenotype that can increase the production of T-cell IFN-γ and protect T cells from activation-induced cell death through the costimulation of 4-1BBL/4-1BB. In addition, the ongoing cross-talk between TANs and activated T cells results the in substantial upregulation of costimulatory molecules on the neutrophil surface, which enhances T-cell proliferation in a positive-feedback loop (89). Although widely accepted, it suffers from some limitations due to the tumors’ progress and the differences between vitro conditions and vivo conditions. In even more advanced tumors, the subpopulations of human TANs likely undergo phenotypic changes and perform different functions. Interestingly, TANs are associated with a poor prognosis in plenty of humans and experimental mouse models. Previous studies showed that there is a negative correlation between CXCL-5 mediated mature neutrophil density and CD8+ T cells in lung tumor nodules. Moreover, neutrophils accumulating in lung tissue contribute to inhibit effector functions in CD8+ T cells (104). Taken together, the complex interaction of TANs with T cells in lung cancer remains complex.

### 3.4 Tumor-associated macrophages

It was found that there were substantial infiltrating macrophages defined as tumor-associated macrophages (TAMs) in tumor tissues. TAMs were considered as the most abundant immune cells in the lung cancer microenvironment. Generally, the cells could be classified into two extremes: antitumor (M1-like) and protumor (M2-like) phenotypes (90). M1-like macrophages are initially activated, and they recruit the CD8+ T cells and NK cells by releasing chemokines and cytokines, resulting in an evoke immune system (90). However, M2-like macrophages could form an immunosuppressive microenvironment, stimulate tumor cell initiation, promote tumor angiogenesis, and subsequently assist tumor metastasis (105). It was reported that M2-like macrophages in the tumor stroma impaired the antitumor activity of CD8+ T cells by secreting STAT3 to the TME and expressing high levels of PD-L1 (90, 106). Platinum therapy increases the differentiation of monocytes to M2-like macrophages that, in turn, leads to platinum resistance (107). It is reported that the complex function between TAMs and cytotoxic T cells is one of the causes of chemotherapy resistance. T-cell immunoglobulin and mucin domain protein-4 (TIM-4) was highly expressed on macrophages, resulting in the reduction of antigen presentation and the impairment of the CTL immune response (108). In addition, M2-like macrophages enhance differentiation toward Tregs and promote the infiltration and function of Tregs, thus facilitating the immune evasion of NSCLC (91).

### 3.5 Low expression of major histocompatibility complex

He et al. assayed the expression of MHC Class II in lung cancer cells and TILs. They found that MHC II was detected both in NSCLC cell lines and tissues. However, they found that MHC II on SCLC cell lines or tissue tumor cells was not expressed. Additionally, MHC Class II expression in SCLC TILs was less than NSCLC TILs (109). In patients with NSCLC, it has been reported that higher MHC Class II is positively related to a better prognosis. Johnson and coworkers found that, in lung adenocarcinoma, cancer cell–specific MHCII (csMHCII) expressed on tumor cells was positively related to T-cell infiltration and retention in the TME (93). Moreover, the expression of MHC Class I is lowest in SCLC compared with other cancer cells (110). The low expression of MHC Class I correlated closely with fewer immune infiltrates in SCLC (92).

### 3.6 CD100

CD100, also called Sema4D, is an important immune semaphorin expressed on T cells (111). There are two forms of CD100: membrane-bound CD100 (mCD100) and soluble CD100 (sCD100). mCD100 is shed from CD8+ T cells and forms sCD100 by matrix metalloproteinases (MMPs) (112, 113). sCD100 plays a significant role in immunoregulatory activity and modulating CD8+ T cells in NSCLC patients. sCD100 enhanced lung-resident and peripheral CD8+ T-cell responses through the interaction between CD27 and CD100 in NSCLC. However, during NSCLC progression, the level of MMP-14 in peripheral blood and the lung-resident microenvironment were decreased. Compared with the healthy control group, the level of serum sCD100 is lower while the level of mCD100 is higher on CD8+ T cells in NSCLC patients. MMP-14 is necessary for the processing and release of sCD100. During the progression of lung cancer, the frequency of serum MMP-14 in the lung-resident microenvironment and peripheral blood is reduced. It is the decreased MMP-14 levels that are insufficient for mCD100 to form sCD100, leading to the lung resident and peripheral CD8+ T-cell inactivation (94).

### 3.7 Commensal microbiota

In 2016, culture-in-dependent sequencing studies have showed that the lower respiratory tract is colonized by a complex diversity of bacteria (114). Multiple lines of clinical evidence have linked the development of lung cancer to increased total bacterial load and altered local microbiota in the lung. Recently, Jin’s group understood these associations mechanistically by a genetically engineered mouse model of LUAD. The result suggested that the dysregulation of local microbiota induced the proliferation and activation of the lung-resident γδT cells. γδT cells that produce IL-17 and other proinflammatory molecules augment the inflammatory response and promote lung cancer proliferation. Overall, this finding indicated that depleting microbiota-induced γδT cells may provide effective and novel therapeutic strategies for the treatment and prevention of lung cancer (23).

### 3.8 Phytochemicals

Phytochemicals are a huge library of natural products that provide anticancer effects against lung cancer. Curcumin is a phenolic substance naturally found in turmeric. Zou et al. reported that curcumin served a significant function in regulating immune responses and Treg types in lung cancer patients. Previous research has showed that Tregs contribute to promote the immunosuppressive TME, allow tumor evasion, and play an important role in lung cancer progression and metastasis. By contrast, it has been pointed out that Th1 is involved in the antitumor activity *via* increasing the expression of type 1 cytokines, such as IFN-γ and IL-2. Th1 plays a role in tumor recognition (115). At the present time, curcumin has been used for cancer control clinical trials. Compared with lung cancer patients treated with placebo for 2 weeks, the frequency of Tregs is lower and the frequency of Th1 cells is higher in the peripheral system of lung cancer patients treated with curcumin for 2 weeks. Researchers isolated Treg cells from the blood samples collected from lung cancer patients, treated them with curcumin for 6 days, and analyzed these cells by flow cytometry. Using this approach, they have been able to demonstrate that curcumin can convert Tregs into Th1 cells in lung cancer patients. The reason for this phenomenon may be explained that curcumin can increase the T-bet promoter activity and the expression of IFN-γ *via* suppressing the Foxp3 activities in Tregs (95). Resveratrol (3,5,4′-trihydroxystilbene, Res) is a well-characterized natural polyphenolic compound found in several food plants, such as peanuts and grapes. Resveratrol is a plant antitoxin antioxidant with antioxidant and anti-inflammatory properties (116). In the recent past, several studies have reported the link between resveratrol and lung cancer, demonstrating its antiproliferative, antimigratory, and proapoptotic properties (117–119). Moreover, some data indicated that resveratrol could enhance chemotherapeutic efficacy as well as display an additive inhibitory effect with cisplatin (120, 121). A previous finding showed that resveratrol could delay tumor progression by reducing granulocytic MDSC (G-MDSC) accumulation, impairing its suppressive ability on CD8+ T cells (96). In an experimental mouse 4T1 tumor model, resveratrol enhanced CD8+ T-cell cytotoxic activity and Th1 immune responses *via* decreasing the expression of PD-1 on pulmonary T cells (97). Due to the wide variety of categories and functions of phytochemicals, future studies are warranted to expound their underlying mechanisms and the potential utility of antitumor therapy.

### 3.9 Metabolic checkpoint

The fate and function of T cells are essentially related to their metabolism, and T cells require metabolic pathways to generate bioenergetic intermediates to the effector function and exertion of antitumor immunity. The activation process of T cells comes with a pronounced increase in metabolism. Naïve T cells maintain low metabolic demand until activated, reserving fuel for those T cells that enter the effector phase. When quiescent T cells encounter the antigen, the first task is to proliferate quickly, demanding a lot of glucose. Effector T cells significantly upregulate the use of oxidative and memory T-cell precursors and become increasingly dependent on mitochondrial to fatty-acid oxidation over time (122). Memory T cells form a fused network of mitochondria under the control of mitochondrial fusion protein optic atrophy 1 (Opa1), which has excellent oxidative function. However, T cells lose mitochondrial activity after entering the TME. CD8+ TIL chronically activates serine/threonine kinase Akt, which inhibits the mitochondrial biogenesis transcriptional coactivator, PGC1α, directly or indirectly *via* Foxo1. The persistent loss of mitochondrial activity and mass leads to the functional defects of CD8+ TIL in turn (123).

The metabolic pathways are also vital to sustain the proliferation and survival of cancer cells. Thus, cancer cells compete with T cells to acquire adequate nutrients, especially glucose, which results in less availability of nutrients to T cells. T cells activated under nutrient-poor conditions will lose their responses to antigen stimulation. The reduction or deprivation of glucose contributes to T-cell dysfunction by limiting aerobic glycolysis and reducing IFN-γ production and mTOR activity (124). The CD28 signaling pathway can facilitate the glucose uptake and metabolism of T cells (125). Immune checkpoint blockades with anti-PD-1 or anti-CTLA-4 mAbs restore the glycolytic capacities and reinvigorate the effector functions of TILs in a mouse tumor model. PD-L1 blockade also directly targets tumor cells to inhibit mTOR activity and decrease the expression of glycolysis enzymes (124). A published study suggested that low glucose availability in the TME constrained the expression of the methyltransferase EZH2 in T cells, limiting effector T-cell polyfunctionality and survival through the miRNA-EZH2-Notch signaling pathway (126). Furthermore, glycolytic metabolite phosphoenolpyruvate (PEP) has been shown to sustain Ca^2+^-NFAT signaling and T-cell activation, while the overexpression of phosphoenolpyruvate carboxy kinase (PCK1) to upregulate PEP-improved T-cell effector functions *via* metabolic reprogramming (127). Of course, the supply of certain amino acids, including arginine, tryptophan, and glutamine, is also essential for T-cell activation and cancer cell proliferation and survival. For example, arginine is lacking in the TME due to the secretion of the arginase 1 by TAMs and MDSCs (128). Moreover, tumor cells lead to excessive glutaminolysis that is required for T-cell activation and proliferation (129). Tumor cells and suppressive immune cells express the tryptophan metabolism enzyme IDO, which decomposes tryptophan in the TME, also resulting in an increase of kynurenine, an immunosuppressive catabolite (130). Accumulated kynurenine, in turn, facilitates regulatory T-cell production (131). Cancer-generated lactic acid is considered a critical immunosuppressive metabolite in the TME. Cancer-generated lactic acid can induce the apoptosis of naïve T cells, which may further promote tumor immune escape due to the loss of the FAK family–interacting protein of 200 kDa (132). Lactic acid induces the PH changes and the loss of cytosolic NAD+ regeneration, which further inhibits T-cell function and cytokine production (133). Therefore, the neutralization of tumor acid has been shown to enhance T-cell antitumor immunity (134). In addition, Treg and tumor cells metabolize extracellular ATP released by the dying cells. The adenosine binds with the adenosine A_2A_ receptor on the surface of T cells to suppress T-cell activation. Hypoxia is another metabolic parameter that is closely linked with rapid tumor growth. Notably, different studies have proven that hypoxia both improves and impairs T cells’ immune responses. Results from murine chronic infections and tumor models indicate that hypoxia can enhance the cytotoxicity of T cells as well as promote viral and tumor clearance (135, 136). By contrast, hypoxia upregulates the expression of inhibitory receptors on T cells and disturbs T-cell functions *via* hypoxia-inducible factor 1α (HIF-1α) (135, 137). A previous finding suggests that therapy with a simultaneous blockade of PD-L1 and the inhibition of HIF-1α may enhance MDSC-mediated T-cell activation (138).

Collectively, the high demand of tumor cells for nutrient leads to the depletion of key nutrients such as glucose and the accumulation of waste products, creating a TME that is metabolically hostile to effector T cells. The TME leads to impaired function of T cells *via* various inhibitory metabolic signalings. Thus, understanding the metabolic properties of tumor cells and the effect of the TME is expected to reactivate dysregulated T cells and synergistically improve existing immunotherapy.

## 4 T-cell-associated cellular immunotherapy for lung cancer

Tumor immunotherapy, which has been developed in recent decades, is thought of as a promising and effective method to treat lung cancer patients. Tumor immunotherapy has been defined as the treatment strategy to combat tumors by generating or augmenting an antitumor immune response against tumor cells (139). T cells are the core of cellular tumor immunotherapy that play an important role in the treatment of lung cancer. In clinical practice, advancements in T-cell-associated cellular immunotherapy have provided plenty of convincing evidence that T-cell-associated cellular immunotherapy is regarded as a method of lung cancer therapy. T-cell-associated cellular immunotherapy affords an opportunity for the study of a complementary method to traditional lung cancer therapeutic therapy.

### 4.1 Adoptive T-cell therapy

**Figure 5:**
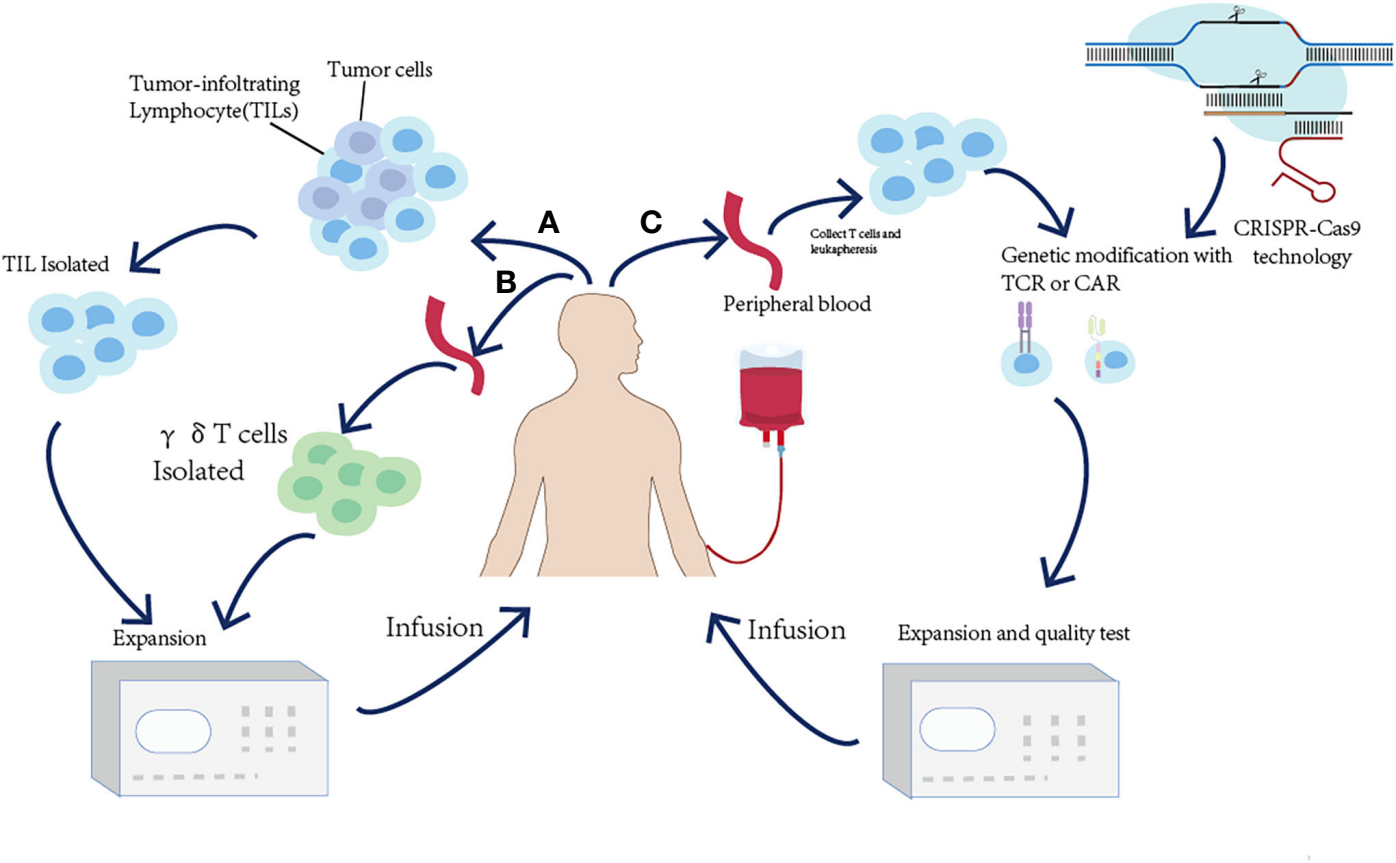
Overview of the adoptive T-cell transfer (ACT) approaches to harness the immune system to treat cancer. **(A)** For TIL ACT, tumor-infiltrating T cells (TILs) are isolated from surgically resected tumor samples and expanded *ex vivo*. **(B)** γδT-cell therapy relies on collecting peripheral blood and expanding γδT cells *ex vivo*. **(C)** For TCR-T or CAR-T ACT, the peripheral blood T cells are isolated and expressed *ex vivo* and genetically modified to express either a TCR or a CAR.

Adoptive T-cell therapy (ACT) is used to expand and infuse natural *ex vivo* manipulated tumor-reactive T cells into the lymphodepleted patients to kill tumor cells by enhancing T-cell responses (140). Based on the divergent strategies utilizing T cells to kill tumors, adoptive T-cell therapy is divided into (i) the isolation of naturally occurring tumor-reactive T cells from existing tumor masses or blood and (ii) the genetic modification of T cells to endow them with the specific recognition of tumor cells. In this part, the basic principles and utilization of ACT will be presented (**Figure 5** and **Table 3**).

**Table 3:** Clinical response to different ACT modalities.

Treatment	Patient no.	Population	Clinical outcome	References
γδT cells	10	IV/rec NSCLC	SD3, PD5	(141)
	15	I-IV NSCLC	SD6, PD6	(142)
	15	Advanced/rec. NSCLC	SD6, PD6	(143)
	10	Advanced NSCLC	SD1, PD1, OS↑	(144)
TIL	56	II-III NSCLC	SR↑	(145)
	11	IIIB NSCLC	SR↑	(146)
	20	Advanced NSCLC	CR2, PR3	(147)
CAR-T	11	IIB-IV NSCLC	PR2, SD5, PD2	(148)
	9	Advanced relapsed/refractory EGFR-positive NSCLC	PR1, SD6, PD2	(149)

SD, stable disease; PD, progressive disease; OS, overall survival; SR, survival rate; ORR, overall response rate; CR, complete response; PR, partial response.

#### 4.1.1 Chimeric antigen receptor-T cell

CAR-T cells are genetically engineered T cells that can recognize and bind with antigens on tumor cells. The general strategy in CAR-T cell is the utilization of gene transfer technology to retargeted patients’ T cells to express a CAR that can bind to common antigens. CARs are artificial fusion proteins that comprise four major components, namely, an intracellular signaling domain, a transmembrane (TM) domain, an extracellular spacer/hinge sequence motif, and an extracellular antigen-binding domain. After the transfection of autologous or allogeneic peripheral blood T cells with the CAR complex, transfected cells are injected into patients as cytotoxic agents, attacking cancer cells (4). In recent years, CAR-T cells have gained the recent success of clinical trials in B-cell hematologic malignancies (150). Two CD19-specific CAR-Ts for certain B-cell malignancy treatments were recently approved by the US Food and Drug Administration (FDA) and the European Medicines Agency (151). At present, one of the tough challenges for researchers in this domain is how to expand CAR-T cell therapy from rare hematologic malignancies to solid tumors including lung cancer. Thus, the efficacy and safety of CAR-T cell therapy in solid cancers, especially lung cancer, are under intensive investigation. As new agents of tumor immunotherapy, CAR-T cells show great promise for the treatment of lung cancer. Finding an ideal target antigen has been one of the greatest obstacles in the field of developing treatment of solid tumors. A suitable target antigen should present highly and selectively on cancer cells with no expression on normal tissues (4, 152). In clinical trials for lung cancer, the commonly candidate targeted antigens include human epidermal growth factor receptor 2 (HER2), inactive tyrosine-protein kinase TM receptor (ROR1), CD80/CD86, PD-L1, carcinoembryonic antigen (CEA), prostate stem cell antigen (PSCA), mucin 1 (MUC1), mesothelin (MSLN), and epidermal growth factor receptor (EGFR) (153). In clinical practice, all of these candidate targeted antigens should be concerned about their safety profile and the extent of on-target and off-tumor effects (154).

#### 4.1.2 T-cell receptor–T cell

TCRs are naturally occurring surface receptors on T cells that can recognize antigens in the context of human leukocyte antigen (HLA) presentation. Typically, the affinity of human TCRs to self-antigens isolated from cancer patients is low due to the impact of local and systemic tolerance on the endogenous antitumor T cells (140). To overcome this issue, the artificially designed high-affinity TCR is encoded in T cells by genetic engineering. The basic principle of TCR-T-cell therapy is to modify the patient’s own T cells *ex vivo* to express tumor antigen-specific TCRs and then expand and reinject them back into the patient’s body to specifically attack malignant tissue (155). The host antitumor immune response selects and activates T cells that recognize tumor antigens. Thus, T cells that obtain TCRs from naturally occurring tumor antigen–specific T (Tas) cells in patients with cancer will target tumor-specific antigens (TSAs) in tumors. Compared with bystander T cells circulating in tissues, Tas cells are characterized by enrichment in tumor, tumor-specific clonal expansion, and neoantigen specificity. Personalized immunotherapy targeting TSAs could induce a highly effective and safe antitumor immune response without damaging normal tissue. T cells engineered with TCRs from these naturally occurring Tas cells in a patient will target personal TSAs in the tumor. However, precisely predicting neoantigens from tumor mutations remains challenging (156). In contrast to the modest success of TCR-T-cell therapy for hematological malignancies, clinical experience in solid tumors including lung cancer is very limited.

#### 4.1.3 Tumor-infiltrating lymphocyte

First isolated from mice tumor samples in 1986 by Rosenberg’ research group, TILs are a cluster of cells involved in innate immune responses that primarily consist of effector T cells and natural killer (NK) cells (140). The quantity of TILs has been proven to positively correlate with the prognosis in lung cancer, making lung cancer an ideal candidate for adoptive cell therapy with TILs (157, 158). TIL therapy involves the extraction and *in vitro* expansion of tumor-specific lymphocyte colonies (159). Now, there has been some clinical evidence suggesting that the efficacy of TIL ACT is promising in lung cancer. In an open-label phase 1 trial from 2021, Creelan et al. assessed the safety and preliminary efficacy of TIL therapy in combination with nivolumab in the nivolumab (a PD-1 inhibitor) monotherapy of advanced-stage NSCLC. These patients received cyclophosphamide and fludarabine lymphodepletion therapy, ACT TIL, and IL-2, followed by maintenance nivolumab. The result suggested that majority of the patients achieve confirmed clinical responses. Among these patients, two achieved complete clinical responses that were ongoing 1.5 years later. The results of the experiment found clear support for the safety and preliminary efficacy of adoptive cell therapy with autologous TILs in lung cancer (147). Overall, although there is still considerably long time needed for TIL ACT to be approved and widely implemented into the clinical practice of lung cancer, much progress in tumor immunology brings hope for this therapy in the future.

#### 4.1.4 γδT cells

γδT cells are a minor subset of T cells characterized by the expression of γδ T-cell receptor, which are not restricted by MHC (160). γδT cells only account for 2%–10% of T lymphocytes in human blood and play a role in immunosurveillance (161). The findings of recent studies reported that γδT cells exert strong cytotoxicity on lung cancer cell lines (162). Recently, Sakamote and colleagues reported that γδT cells stimulated by zoledronate can recognize and kill the NCI-H358 lung cancer cell line (142). At present, the safety and efficacy of γδT cell therapy are being evaluated in prospective clinical trials. The clinical trials response is promising and worth further studying.

#### 4.1.5 Clustered regularly interspaced short palindromic repeat–engineered T cells

Although adoptive T-cell therapy is a novel and promising approach for antitumors, it has some drawbacks since its introduction, including limitations in hematological malignancies, insertional oncogenesis, T-cell exhaustion and durability, the risk of graft-versus-host disease (GVHD), and manufacturing costs (163). The emergence of clustered regularly interspaced short palindromic repeat (CRISPR)-Cas9 technology reinvigorated tumor immunotherapy as a tool for further realizing the potential of adoptive T-cell therapy. CRISPR and its associated proteins (Cas) are vital components of the crucially adaptive immune system, which exist in archaea, bacteria, and mitochondria. Currently, the CRISPR-Cas9 system has been engineered into a precise and efficient genome editing tool and widely used to edit genomes (164). Briefly, the CRISPR-Cas9 technology edits the target DNA help Cas9 recognize the protospacer-adjacent motif (PAM) upstream or downstream of the target sequence by using a single-guide RNA (gRNA) and then induces the creation of the double-strand breaks (DSBs) of the target DNA (165). Since the Cas9 DNA endonuclease enzyme can target any site in the genome and create DSBs, multiplex and precise gene editing can be achieved with as little unnecessary as possible. CRISPR-Cas9 technology can edit genes at multiple loci possible simultaneously, allowing for the improvement of the time and process frame of gene editing (164). On the one hand, CRISPR-Cas9 technology is used to improve efficacy and expands the application range of CAR-T cells. On the other hand, it is also widely used to modify immune cells including T cells to enhance the antitumor effect. The world-first phase I clinical trial was initiated to investigate the safety and feasibility of CRISPR-Cas9 PD-1-edited T-cell therapy in advanced NSCLC. A total of 22 advanced NSCLC patients whose disease had failed after multiple lines of anticancer therapy were enrolled. A total of 17 patients among them had sufficient edited T cells for reinfusion. The results demonstrated the general safety and feasibility of CRISPR-Cas9 gene–edited T cells in lung cancer (166). Although the application of CRISPR-Cas9 technology still faced a lot of problems, such as PAM dependence, off-target mutations, delivery methods, and sgRNA production, there is no doubt that this technology will play an important role in the development of the next generation of adoptive T-cell technology (164).

### 4.2 Immune checkpoint inhibitors

**Figure 6:**
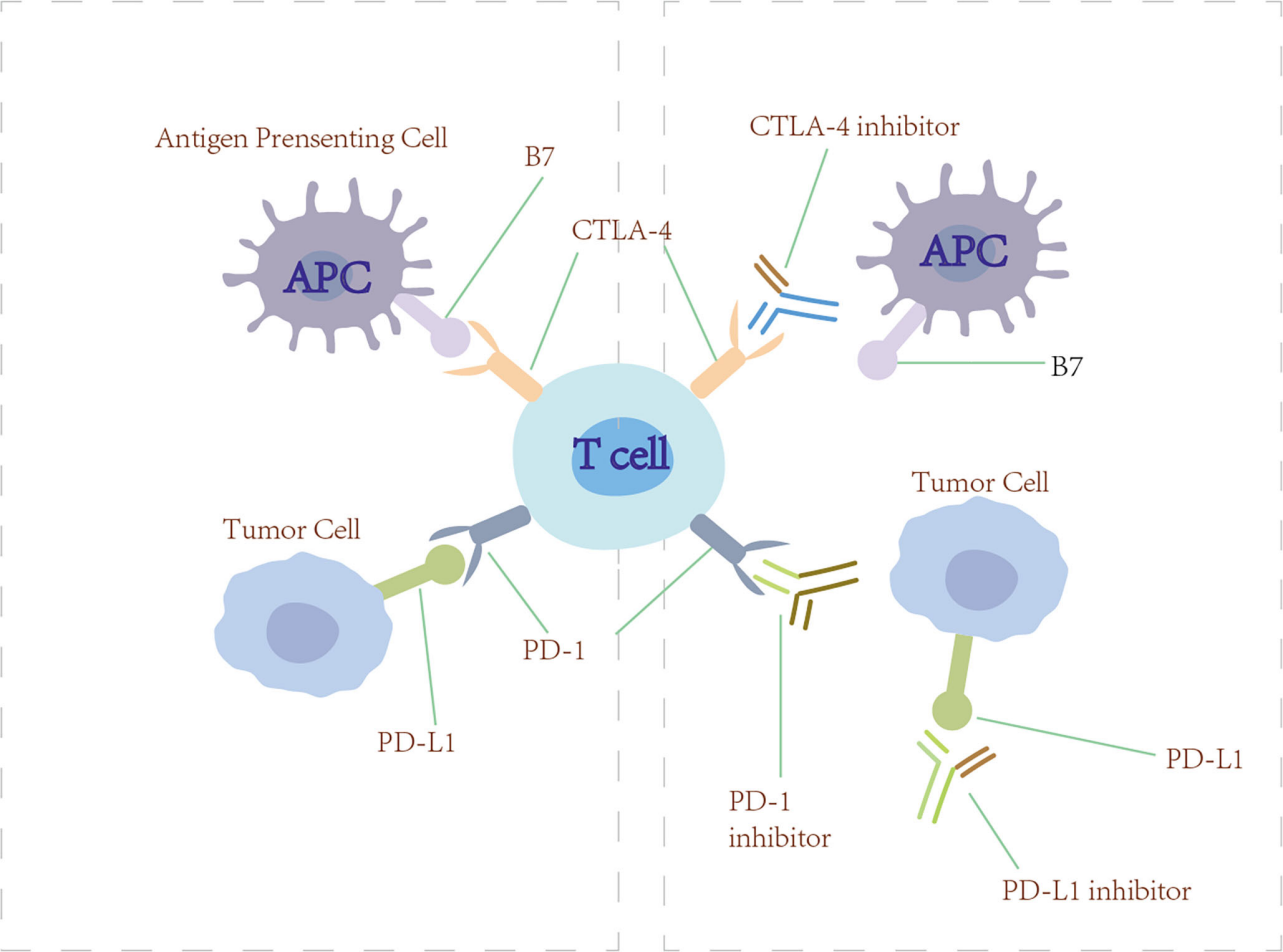
Checkpoint inhibitors. Anti-PD-1/PD-L1 and anti-CTLA-4 (checkpoint inhibitors) block PD-1/PD-L1 and CTLA-4 from engaging their ligands, thus restoring the ability of T cells to recognize tumor and initiate immunotherapy.

Tumor cells are able to release various immunosuppressive factors. Meanwhile, corresponding immune inhibitory receptors on cytotoxic T lymphocytes bind with them. This function mechanisms activate downstream signal transduction pathways to restrain the cytokine secretion of cytotoxic T lymphocytes and greatly restrain the immune function of tumor-specific T lymphocytes. These coinhibitory receptors could reduce tumor-specific CD4+ and CD8+ T-cell effector function, impair T-cell functionality, and induce T-cell exhaustion on a systemic level that leads to generalized immune suppression (167). Now, we are seeing great potential for treatment in this area (**Figure 6**).

#### 4.2.1 Programmed cell death protein 1/programmed cell death ligand 1 blockade immunotherapy

In the normal immune process, the PD-1/PD-L1 pathway restrains the proliferation of T cells and protects humans from injury resulting from autoimmune disease and excessively strong immunoreactions. However, some studies have found that the PD-L1 that expressed on the tumor cells binds with the PD-1 receptor on T cells, leading to a negative regulation of T cells and immunological escape in tumors (139). The basic mechanism of PD-1/PD-L1 blockade immunotherapy is to restore the ability of T cells to recognize tumors and initiate immunotherapy by blocking the PD-1 receptor or PD-L1 ligand. Currently, the monoclonal anti-PD-1/PD-L1 antibodies that were approved by the US FDA to treat advanced NSCLC include nivolumab, pembrolizumab, durvalumab, atezolizumab, and avelumab (168). In KEYNOTE-189, 616 patients with metastatic non-squamous NSCLC were randomized (1:2) to receive pemetrexed and platinum plus pembrolizumab or placebo every 3 weeks for four cycles. OS and PFS were significantly longer compared with placebo plus pemetrexed-platinum (169).

#### 4.2.2 Cytotoxic T-lymphocyte-associated antigen 4 blockade immunotherapy

CTLA-4 is an immune checkpoint receptor found on the effector and regulatory T cells (170). Normally, CTLA-4 is upregulated on the cell surface where it functions to mediate the inhibitory signaling of T cells through outcompeting CD28 for binding to its ligand B7 and inducing T-cell cycle arrest. Through these mechanisms, CTLA-4 has a crucial role in maintaining normal immunologic homeostasis as evidenced by the fact that mouse models lacking CTLA-4 die from fatal lymphoproliferation (171). Initial preclinical proof-of-principle studies showed that the antibody blockade of CTLA-4 could lead to antitumor immunity in syngeneic animal models (172). Tremelimumab and ipilimub are therapeutic antibodies targeting CTLA-4 (170). However, CTLA-4 inhibitors have shown minimal single-agent activity in lung cancer patients. Interestingly, the use of ipilimumab in combination with cytotoxic chemotherapy has shown more promising results. In a randomized, double-blind, multicenter phase II study, Lynch et al. evaluated the efficacy of the use of ipilimumab in combination with paclitaxel and carboplatin as first-line treatment in stage IIIB/IV NSCLC. The results showed that phased ipilimumab plus paclitaxel and carboplatin significantly improved irPFS and PFS (173).

### 4.3 Non-classical T-cell-associated cellular immunotherapy

In addition to the common treatment modalities described above, there are still some non-classical T-cell-related therapies that deserve attention.

The upregulation of angiogenesis in lung cancer is a frequent occurrence that is reported to predict a negative prognosis. Thus, antiangiogenic agents are projected to be a promising strategy for cancer treatment (174). Previous studies showed that antiangiogenic agents combined with anti-PD-L1 stimulate the activation and infiltration of T cells. The VEGF/VEGFR inhibitors have a regulatory effect on the immune status of tumors by enhancing T-cell infiltration and activation. In addition, CD8+ T cells isolated from responding tumors expressed elevated granzyme B protein and perforin mRNA levels (175). In an autochthonous mouse model of SCLC, the treatment outcome of mice treated with combined anti-PD-L1 and anti-VEGF targeted therapy is significantly successful compared to anti-VEGF and anti-PD-L1 monotherapy. Researchers further analyzed that T cells isolated from the tumors of mice bearing SCLC, CD4+ TILs, and CD8+ T TILs were observed to infiltrate the tumor, while few FOXP3+ TITLs gather in the tumor bed (176).

In addition to VEGF/VEGFR inhibitors, the intrafocal injection of an oncolytic virus (OV) can effectively infect tumor cells and induce tumor lysis, promoting a tumor-specific immune response by inducing the CD8+ T-cell infiltration and upregulation of immune-related gene signatures. Specially, OVs propagate in cancer cells and specially target and kill them but not healthy cells (177). In 2015, the FDA approved the first immunotherapy oncolytic virus for the treatment of melanoma, which has invigorated the field. In 2019, Kellish et al. found a modified oncolytic myxoma virus (MYXV) that shows tumor-specific cytotoxicity efficiently in SCLC. In immunocompetent SCLC mouse models, the OS of mice treated with MYXV was significantly prolonged. Meanwhile, they observed that the CD45+ lymphocyte infiltration is associated with tumor necrosis and growth inhibition in syngeneic murine allograft tumors (178). A randomized double-blind phase II trial evaluated the Seneca Valley virus (NTX-010) in patients with ED-SCLC after chemotherapy with a platinum doublet. However, the result showed that these patients did not benefit from NTX-010 treatment and the trial was terminated attributing to futility (179). At present, the level of clinical evidence in OV therapy is still scarce and needs to be further studied in the future.

TLF is a recombinant human lactoferrin. Lactoferrin, an iron-binding glycoprotein of the innate immune defense, is a member of the transferrin family of non-heme iron-binding proteins. Lactoferrin has been proven to potentiate an adaptive immune response that can stimulate the activation of tumor antigen-bearing DCs and CD8+ T cells (180). In a randomized, double-blind, placebo-controlled phase II trial, 100 stage III/IV NSCLC patients whose disease had failed after one or two lines of systemic anticancer therapy were randomly to receive either oral TLF (1.5 g twice per day) or placebo (15 ml twice per day) in addition to supportive care. OS has apparent improvement in patients who received TLF. A trend toward the extension was also observed in PFS and the disease control rate (181). An international, multicenter, randomized, double-blind study was initiated to investigate talactoferrin alfa in advanced NSCLC patients who progressed after two or more previous regimens. The primary endpoint was OS. However, the results observed in NSCLS patients treated with talactoferrin alfa were disappointing. Based on these results, further studies are needed to understand whether TLF could be considered a promising therapy to treat lung cancer (182).

Finally, IL-2 is a T-cell growth factor that can enhance the immune response of the tumor by facilitating the activation of T cells. A study with IL-2 plus MLT in patients with advanced cancer has demonstrated partial response and stable disease in 4 out of 20 (20%) and 10 out of 20 (50%) patients, respectively (183). However, in a phase III trial, Ridolh et al. report no response in 241 advanced NSCLC patients treated with subcutaneous low dose IL-2 and first-line chemotherapy with cisplatin and gemcitabine (184). In the future, more studies are needed to acknowledge the efficacy of IL-2 and explore the strategy of combined therapy.

## 5 Discussion

The present article gives an overview of the tumor-reactive T-cell biological functions and prognostic value in lung cancer patients and sums up the affecting factors and therapeutic strategies associated with T cells. Lung cancer is a challenging disease in the need of new therapeutic opportunities. Immunotherapy has swiftly risen to become one of the major pillars in cancer treatment. The use of immunotherapy can benefit lung cancer patients by generating effective antitumor responses in the host. T cells have been observed to influence the genesis, progression, and metastasis of lung cancer through cell interaction–dependent and cytokine-mediated effects. T cells are considered as one of the promising therapeutic target candidates as T cells are a critical component of our immune system despite the fact that T-cell associated cellular immunotherapy is not suitable for everyone and the current clinical response rate is not satisfactory. However, an increasing understanding of tumor molecular biology and the immune system has led to new possibilities in treatment strategies. Although it may not be a curative treatment for lung cancer, T-cell immunotherapy can effectively remove residual tumor cells after resection in early-stage lung cancer patients.

The biological characteristics of tumor cells determine the diversity and complexity of antitumor mechanisms. In clinical practice, T-cell-associated cellular immunotherapy in combination with traditional treatment methods is expected to become a measure to improve OS in lung cancer patients. The results of ongoing large randomized trials along with future research and novel immunotherapies and new combinations are expected to define the final role of immunotherapy in the treatment algorithm for lung cancer. Identifying the functions and regulations of the T cells associated with the development and metastasis of lung cancer and explore how these T cells execute effector function will be crucial to explore a number of new T-cell-oriented immunotherapeutic strategies.

## Author contributions

Writing—original draft, YW; Validation, MY and CW; Investigation, YC; Writing—review and editing, JZ and YZ; Funding acquisition, JZ and YZ. All authors have read and agreed to the published version of the manuscript.

## References

[1] Oliver AL. Lung cancer: Epidemiology and screening. Surg Clin North Am (2022) 102(3):335–44. doi: 10.1016/j.suc.2021.12.001 35671760

[2] Lu T Yang X Huang Y Zhao M Li M Ma K. Trends in the incidence, treatment, and survival of patients with lung cancer in the last four decades. Cancer Manag Res (2019) 11:943–53. doi: 10.2147/CMAR.S187317 PMC6345192 30718965

[3] Kaur J Elms J Munn AL Good D Wei MQ. Immunotherapy for non-small cell lung cancer (Nsclc), as a stand-alone and in combination therapy. Crit Rev Oncol Hematol (2021) 164:103417. doi: 10.1016/j.critrevonc.2021.103417 34242772

[4] Xiao BF Zhang JT Zhu YG Cui XR Lu ZM Yu BT. Chimeric antigen receptor T-cell therapy in lung cancer: Potential and challenges. Front Immunol (2021) 12:782775. doi: 10.3389/fimmu.2021.782775 34790207 PMC8591168

[5] Ren S Xiong X You H Shen J Zhou P. The combination of immune checkpoint blockade and angiogenesis inhibitors in the treatment of advanced non-small cell lung cancer. Front Immunol (2021) 12:689132. doi: 10.3389/fimmu.2021.689132 34149730 PMC8206805

[6] Quaratino S Forssmann U Marschner JP. New approaches in immunotherapy for the treatment of lung cancer. Curr Top Microbiol Immunol (2017) 405:1–31. doi: 10.1007/82_2014_428 25522903

[7] Kumar BV Connors TJ Farber DL. Human T cell development, localization, and function throughout life. Immunity (2018) 48(2):202–13. doi: 10.1016/j.immuni.2018.01.007 PMC5826622 29466753

[8] Zhang M Zhang S. T Cells in fibrosis and fibrotic diseases. Front Immunol (2020) 11:1142. doi: 10.3389/fimmu.2020.01142 32676074 PMC7333347

[9] Kaminski H Couzi L Eberl M. Unconventional T cells and kidney disease. Nat Rev Nephrol (2021) 17(12):795–813. doi: 10.1038/s41581-021-00466-8 34446934

[10] Wingender G Kronenberg M. Omip-030: Characterization of human T cell subsets *Via* surface markers. Cytometry A (2015) 87(12):1067–9. doi: 10.1002/cyto.a.22788 26506224

[11] Blom B Spits H. Development of human lymphoid cells. Annu Rev Immunol (2006) 24:287–320. doi: 10.1146/annurev.immunol.24.021605.090612 16551251

[12] Barua S Fang P Sharma A Fujimoto J Wistuba I Rao AUK. Spatial interaction of tumor cells and regulatory T cells correlates with survival in non-small cell lung cancer. Lung Cancer (2018) 117:73–9. doi: 10.1016/j.lungcan.2018.01.022 PMC6294443 29409671

[13] Kwiecien I Stelmaszczyk-Emmel A Polubiec-Kownacka M Dziedzic D Domagala-Kulawik J. Elevated regulatory T cells, surface and intracellular ctla-4 expression and interleukin-17 in the lung cancer microenvironment in humans. Cancer Immunol Immunother (2017) 66(2):161–70. doi: 10.1007/s00262-016-1930-6 PMC5281670 27866241

[14] Budna J Spychalski L Kaczmarek M Frydrychowicz M Gozdzik-Spychalska J Batura-Gabryel H. Regulatory T cells in malignant pleural effusions subsequent to lung carcinoma and their impact on the course of the disease. Immunobiology (2017) 222(3):499–505. doi: 10.1016/j.imbio.2016.10.017 27773662

[15] Erfani N Mehrabadi SM Ghayumi MA Haghshenas MR Mojtahedi Z Ghaderi A. Increase of regulatory T cells in metastatic stage and ctla-4 over expression in lymphocytes of patients with non-small cell lung cancer (Nsclc). Lung Cancer (2012) 77(2):306–11. doi: 10.1016/j.lungcan.2012.04.011 22608141

[16] Marshall EA Ng KW Kung SH Conway EM Martinez VD Halvorsen EC. Emerging roles of T helper 17 and regulatory T cells in lung cancer progression and metastasis. Mol Cancer (2016) 15(1):67. doi: 10.1186/s12943-016-0551-1 27784305 PMC5082389

[17] Zhang D Chen Z Wang DC Wang X. Regulatory T cells and potential inmmunotherapeutic targets in lung cancer. Cancer Metastasis Rev (2015) 34(2):277–90. doi: 10.1007/s10555-015-9566-0 25962964

[18] Carmi Y Rinott G Dotan S Elkabets M Rider P Voronov E. Microenvironment-derived il-1 and il-17 interact in the control of lung metastasis. J Immunol (2011) 186(6):3462–71. doi: 10.4049/jimmunol.1002901 21300825

[19] Mateu-Jimenez M Curull V Pijuan L Sanchez-Font A Rivera-Ramos H Rodriguez-Fuster A. Systemic and tumor Th1 and Th2 inflammatory profile and macrophages in lung cancer: Influence of underlying chronic respiratory disease. J Thorac Oncol (2017) 12(2):235–48. doi: 10.1016/j.jtho.2016.09.137 27793775

[20] Heim L Yang Z Tausche P Hohenberger K Chiriac MT Koelle J. Il-9 producing tumor-infiltrating lymphocytes and treg subsets drive immune escape of tumor cells in non-small cell lung cancer. Front Immunol (2022) 13:859738. doi: 10.3389/fimmu.2022.859738 35514957 PMC9065342

[21] Wang W Hodkinson P McLaren F MacKinnon A Wallace W Howie S. Small cell lung cancer tumour cells induce regulatory T lymphocytes, and patient survival correlates negatively with Foxp3+ cells in tumour infiltrate. Int J Cancer (2012) 131(6):E928–37. doi: 10.1002/ijc.27613 22532287

[22] Li Q Han Y Fei G Guo Z Ren T Liu Z. Il-17 promoted metastasis of non-Small-Cell lung cancer cells. Immunol Lett (2012) 148(2):144–50. doi: 10.1016/j.imlet.2012.10.011 23089548

[23] Jin C Lagoudas GK Zhao C Bullman S Bhutkar A Hu B. Commensal microbiota promote lung cancer development *via* gammadelta T cells. Cell (2019) 176(5):998–1013 e16. doi: 10.1016/j.cell.2018.12.040 30712876 PMC6691977

[24] Cui C Wang J Fagerberg E Chen PM Connolly KA Damo M. Neoantigen-driven b cell and Cd4 T follicular helper cell collaboration promotes anti-tumor Cd8 T cell responses. Cell (2021) 184(25):6101–18 e13. doi: 10.1016/j.cell.2021.11.007 34852236 PMC8671355

[25] Zeng R Spolski R Finkelstein SE Oh S Kovanen PE Hinrichs CS. Synergy of il-21 and il-15 in regulating Cd8+ T cell expansion and function. J Exp Med (2005) 201(1):139–48. doi: 10.1084/jem.20041057 PMC2212766 15630141

[26] Sondergaard H Galsgaard ED Bartholomaeussen M Straten PT Odum N Skak K. Intratumoral interleukin-21 increases antitumor immunity, tumor-infiltrating Cd8+ T-cell density and activity, and enlarges draining lymph nodes. J Immunother (2010) 33(3):236–49. doi: 10.1097/CJI.0b013e3181c0c1cb 20445344

[27] Zhang W Chen Y Wei H Zheng C Sun R Zhang J. Antiapoptotic activity of autocrine interleukin-22 and therapeutic effects of interleukin-22-Small interfering rna on human lung cancer xenografts. Clin Cancer Res (2008) 14(20):6432–9. doi: 10.1158/1078-0432.CCR-07-4401 18927282

[28] Niu Y Ye L Peng W Wang Z Wei X Wang X. Il-26 promotes the pathogenesis of malignant pleural effusion by enhancing Cd4(+) il-22(+) T-cell differentiation and inhibiting Cd8(+) T-cell cytotoxicity. J Leukoc Biol (2021) 110(1):39–52. doi: 10.1002/JLB.1MA0221-479RR 33847412

[29] Kinoshita F Tagawa T Akamine T Takada K Yamada Y Oku Y. Interleukin-38 promotes tumor growth through regulation of Cd8(+) tumor-infiltrating lymphocytes in lung cancer tumor microenvironment. Cancer Immunol Immunother (2021) 70(1):123–35. doi: 10.1007/s00262-020-02659-9 PMC10991933 32653939

[30] Hodge G Barnawi J Jurisevic C Moffat D Holmes M Reynolds PN. Lung cancer is associated with decreased expression of perforin, granzyme b and interferon (Ifn)-gamma by infiltrating lung tissue T cells, natural killer (Nk) T-like and nk cells. Clin Exp Immunol (2014) 178(1):79–85. doi: 10.1111/cei.12392 24894428 PMC4360197

[31] Neurath MF Finotto S. The emerging role of T cell cytokines in non-small cell lung cancer. Cytokine Growth Factor Rev (2012) 23(6):315–22. doi: 10.1016/j.cytogfr.2012.08.009 23022528

[32] Ganesan AP Johansson M Ruffell B Yagui-Beltran A Lau J Jablons DM. Tumor-infiltrating regulatory T cells inhibit endogenous cytotoxic T cell responses to lung adenocarcinoma. J Immunol (2013) 191(4):2009–17. doi: 10.4049/jimmunol.1301317 PMC3774528 23851682

[33] Park JH Ko JS Shin Y Cho JY Oh HA Bothwell AL. Intranuclear interactomic inhibition of Foxp3 suppresses functions of treg cells. Biochem Biophys Res Commun (2014) 451(1):1–7. doi: 10.1016/j.bbrc.2014.06.141 25044110

[34] Peng J Yu Z Xue L Wang J Li J Liu D. The effect of Foxp3-overexpressing treg cells on non-small cell lung cancer cells. Mol Med Rep (2018) 17(4):5860–8. doi: 10.3892/mmr.2018.8606 PMC5866031 29436663

[35] Zhang LN Xin T Chen M Gao P. Chemoresistance in mesenchymal lung cancer cells is correlated to high regulatory T cell presence in the tumor microenvironment. IUBMB Life (2019) 71(7):986–91. doi: 10.1002/iub.2043 31066485

[36] Domvri K Petanidis S Zarogoulidis P Anestakis D Tsavlis D Bai C. Treg-dependent immunosuppression triggers effector T cell dysfunction *Via* the Sting/Ilc2 axis. Clin Immunol (2021) 222:108620. doi: 10.1016/j.clim.2020.108620 33176208

[37] Liang J Tian C Zeng Y Yang Q Liu Y Liu Y. Foxa1(+) regulatory T cells: A novel T cell subset that suppresses antitumor immunity in lung cancer. Biochem Biophys Res Commun (2019) 514(1):308–15. doi: 10.1016/j.bbrc.2019.04.152 31036318

[38] Haruna M Ueyama A Yamamoto Y Hirata M Goto K Yoshida H. The impact of Ccr8+ regulatory T cells on cytotoxic T cell function in human lung cancer. Sci Rep (2022) 12(1):5377. doi: 10.1038/s41598-022-09458-5 35354899 PMC8967908

[39] Hughes CE Nibbs RJB. A guide to chemokines and their receptors. FEBS J (2018) 285(16):2944–71. doi: 10.1111/febs.14466 PMC6120486 29637711

[40] Zhu J. T Helper cell differentiation, heterogeneity, and plasticity. Cold Spring Harb Perspect Biol (2018) 10(10):a030338. doi: 10.1101/cshperspect.a030338 28847903 PMC6169815

[41] Yi FS Zhai K Shi HZ. Helper T cells in malignant pleural effusion. Cancer Lett (2021) 500:21–8. doi: 10.1016/j.canlet.2020.12.016 33309856

[42] Wang J Zhou J Zhou Q Qi Y Zhang P Yan C. Dysregulated Th1 cells in lung squamous cell carcinoma. J Leukoc Biol (2022) 112(6):1567–76. doi: 10.1002/JLB.1MA0422-208R 35686499

[43] Ito N Nakamura H Metsugi H Ohgi S. Dissociation between T helper type 1 and type 2 differentiation and cytokine production in tumor-infiltrating lymphocytes in patients with lung cancer. Surg Today (2001) 31(5):390–4. doi: 10.1007/s005950170127 11381500

[44] Salazar Y Zheng X Brunn D Raifer H Picard F Zhang Y. Microenvironmental Th9 and Th17 lymphocytes induce metastatic spreading in lung cancer. J Clin Invest (2020) 130(7):3560–75. doi: 10.1172/JCI124037 PMC7324180 32229721

[45] Dardalhon V Awasthi A Kwon H Galileos G Gao W Sobel RA. Il-4 inhibits tgf-Beta-Induced Foxp3+ T cells and, together with tgf-beta, generates il-9+ il-10+ Foxp3(-) effector T cells. Nat Immunol (2008) 9(12):1347–55. doi: 10.1038/ni.1677 PMC2999006 18997793

[46] Xu C Hao K Yu L Zhang X. Serum interleukin-17 as a diagnostic and prognostic marker for non-small cell lung cancer. Biomarkers (2014) 19(4):287–90. doi: 10.3109/1354750X.2014.908954 24731052

[47] Chen X Wan J Liu J Xie W Diao X Xu J. Increased il-17-Producing cells correlate with poor survival and lymphangiogenesis in nsclc patients. Lung Cancer (2010) 69(3):348–54. doi: 10.1016/j.lungcan.2009.11.013 20022135

[48] Nieto JC Zamora C Porcel JM Mulet M Pajares V Munoz-Fernandez AM. Migrated T lymphocytes into malignant pleural effusions: An indicator of good prognosis in lung adenocarcinoma patients. Sci Rep (2019) 9(1):2996. doi: 10.1038/s41598-018-35840-3 30816121 PMC6395746

[49] Lim C Savan R. The role of the il-22/Il-22r1 axis in cancer. Cytokine Growth Factor Rev (2014) 25(3):257–71. doi: 10.1016/j.cytogfr.2014.04.005 24856143

[50] Zou W Restifo NP. T(H)17 cells in tumour immunity and immunotherapy. Nat Rev Immunol (2010) 10(4):248–56. doi: 10.1038/nri2742 PMC3242804 20336152

[51] Ye ZJ Zhou Q Gu YY Qin SM Ma WL Xin JB. Generation and differentiation of il-17-Producing Cd4+ T cells in malignant pleural effusion. J Immunol (2010) 185(10):6348–54. doi: 10.4049/jimmunol.1001728 20952674

[52] Lee GR. The balance of Th17 versus treg cells in autoimmunity. Int J Mol Sci (2018) 19(3):730. doi: 10.3390/ijms19030730 29510522 PMC5877591

[53] Duan MC Han W Jin PW Wei YP Wei Q Zhang LM. Disturbed Th17/Treg balance in patients with non-small cell lung cancer. Inflammation (2015) 38(6):2156–65. doi: 10.1007/s10753-015-0198-x 26077695

[54] Zhao L Yang J Wang H-P Liu R-Y. Imbalance in the Th17/Treg and cytokine environment in peripheral blood of patients with adenocarcinoma and squamous cell carcinoma. Med Oncol (2013) 30(1):461. doi: 10.1007/s12032-013-0461-7 23335103

[55] Guo Z Liang H Xu Y Liu L Ren X Zhang S. The role of circulating T follicular helper cells and regulatory cells in non-small cell lung cancer patients. Scand J Immunol (2017) 86(2):107–12. doi: 10.1111/sji.12566 28513867

[56] Ma QY Huang DY Zhang HJ Chen J Miller W Chen XF. Function of follicular helper T cell is impaired and correlates with survival time in non-small cell lung cancer. Int Immunopharmacol (2016) 41:1–7. doi: 10.1016/j.intimp.2016.10.014 27788370

[57] Farhood B Najafi M Mortezaee K. Cd8(+) cytotoxic T lymphocytes in cancer immunotherapy: A review. J Cell Physiol (2019) 234(6):8509–21. doi: 10.1002/jcp.27782 30520029

[58] Sun Y Zhai C Chen X Dong Z Hou L Zhou C. Characterization of pd-L1 protein expression and Cd8(+) tumor-infiltrating lymphocyte density, and their associations with clinical outcome in small-cell lung cancer. Transl Lung Cancer Res (2019) 8(6):748–59. doi: 10.21037/tlcr.2019.10.09 PMC6976348 32010554

[59] Brambilla E Le Teuff G Marguet S Lantuejoul S Dunant A Graziano S. Prognostic effect of tumor lymphocytic infiltration in resectable non-Small-Cell lung cancer. J Clin Oncol (2016) 34(11):1223–30. doi: 10.1200/JCO.2015.63.0970 PMC4872323 26834066

[60] Ruffini E Asioli S Filosso PL Lyberis P Bruna MC Macrì L. Clinical significance of tumor-infiltrating lymphocytes in lung neoplasms. Ann Thorac Surg (2009) 87(2):365–72. doi: 10.1016/j.athoracsur.2008.10.067 19161739

[61] Bonanno L Pavan A Dieci MV Di Liso E Schiavon M Comacchio G. The role of immune microenvironment in small-cell lung cancer: Distribution of pd-L1 expression and prognostic role of Foxp3-positive tumour infiltrating lymphocytes. Eur J Cancer (2018) 101:191–200. doi: 10.1016/j.ejca.2018.06.023 30077124

[62] Wang H Li Z Dong B Sun W Yang X Liu R. Prognostic significance of pd-L1 expression and Cd8+ T cell infiltration in pulmonary neuroendocrine tumors. Diagn Pathol (2018) 13(1):30. doi: 10.1186/s13000-018-0712-1 29789013 PMC5964902

[63] Lopez de Rodas M Nagineni V Ravi A Datar IJ Mino-Kenudson M Corredor G. Role of tumor infiltrating lymphocytes and spatial immune heterogeneity in sensitivity to pd-1 axis blockers in non-small cell lung cancer. J Immunother Cancer (2022) 10(6):e004440. doi: 10.1136/jitc-2021-004440 35649657 PMC9161072

[64] Corredor G Wang X Zhou Y Lu C Fu P Syrigos K. Spatial architecture and arrangement of tumor-infiltrating lymphocytes for predicting likelihood of recurrence in early-stage non-small cell lung cancer. Clin Cancer Res (2019) 25(5):1526–34. doi: 10.1158/1078-0432.CCR-18-2013 PMC6397708 30201760

[65] Obeid JM Wages NA Hu Y Deacon DH Slingluff CL Jr. Heterogeneity of Cd8(+) tumor-infiltrating lymphocytes in non-Small-Cell lung cancer: Impact on patient prognostic assessments and comparison of quantification by different sampling strategies. Cancer Immunol Immunother (2017) 66(1):33–43. doi: 10.1007/s00262-016-1908-4 27770170 PMC5512540

[66] Trojan A Urosevic M Dummer R Nestle FO Stahel RA. Real-time polymerase chain reaction monitoring of epithelial cell adhesion molecule-induced T-cell stimulation in patients with lung cancer and healthy individuals using lightcycler technology. J Immunother (2002) 25(3):264–8. doi: 10.1097/00002371-200205000-00009 12000868

[67] Zhang Z Lu M Qin Y Gao W Tao L Su W. Neoantigen: A new breakthrough in tumor immunotherapy. Front Immunol (2021) 12:672356. doi: 10.3389/fimmu.2021.672356 33936118 PMC8085349

[68] Prado-Garcia H Romero-Garcia S Aguilar-Cazares D Meneses-Flores M Lopez-Gonzalez JS. Tumor-induced Cd8+ T-cell dysfunction in lung cancer patients. Clin Dev Immunol (2012) 2012:741741. doi: 10.1155/2012/741741 23118782 PMC3483679

[69] Xu L Chen D Lu C Liu X Wu G Zhang Y. Advanced lung cancer is associated with decreased expression of perforin, Cd95, Cd38 by circulating Cd3+Cd8+ T lymphocytes. Ann Clin Lab Sci (2015) 45(5):528–32. 26586704

[70] Genova C Dellepiane C Carrega P Sommariva S Ferlazzo G Pronzato P. Therapeutic implications of tumor microenvironment in lung cancer: Focus on immune checkpoint blockade. Front Immunol (2021) 12:799455. doi: 10.3389/fimmu.2021.799455 35069581 PMC8777268

[71] Prado-Garcia H Romero-Garcia S Morales-Fuentes J Aguilar-Cazares D Lopez-Gonzalez JS. Activation-induced cell death of memory Cd8+ T cells from pleural effusion of lung cancer patients is mediated by the type ii fas-induced apoptotic pathway. Cancer Immunol Immunother (2012) 61(7):1065–80. doi: 10.1007/s00262-011-1165-5 PMC11028981 22159518

[72] Thommen DS Schumacher TN. T Cell dysfunction in cancer. Cancer Cell (2018) 33(4):547–62. doi: 10.1016/j.ccell.2018.03.012 PMC7116508 29634943

[73] Schietinger A Greenberg PD. Tolerance and exhaustion: Defining mechanisms of T cell dysfunction. Trends Immunol (2014) 35(2):51–60. doi: 10.1016/j.it.2013.10.001 24210163 PMC3946600

[74] Xia A Zhang Y Xu J Yin T Lu XJ. T Cell dysfunction in cancer immunity and immunotherapy. Front Immunol (2019) 10:1719. doi: 10.3389/fimmu.2019.01719 31379886 PMC6659036

[75] Zarour HM. Reversing T-cell dysfunction and exhaustion in cancer. Clin Cancer Res (2016) 22(8):1856–64. doi: 10.1158/1078-0432.CCR-15-1849 PMC4872712 27084739

[76] Thommen DS Schreiner J Muller P Herzig P Roller A Belousov A. Progression of lung cancer is associated with increased dysfunction of T cells defined by coexpression of multiple inhibitory receptors. Cancer Immunol Res (2015) 3(12):1344–55. doi: 10.1158/2326-6066.CIR-15-0097 26253731

[77] Schreiner J Thommen DS Herzig P Bacac M Klein C Roller A. Expression of inhibitory receptors on intratumoral T cells modulates the activity of a T cell-bispecific antibody targeting folate receptor. Oncoimmunology (2016) 5(2):e1062969. doi: 10.1080/2162402X.2015.1062969 27057429 PMC4801463

[78] Guo L Li X Liu R Chen Y Ren C Du S. Tox correlates with prognosis, immune infiltration, and T cells exhaustion in lung adenocarcinoma. Cancer Med (2020) 9(18):6694–709. doi: 10.1002/cam4.3324 PMC7520261 32700817

[79] Scott AC Dundar F Zumbo P Chandran SS Klebanoff CA Shakiba M. Tox is a critical regulator of tumour-specific T cell differentiation. Nature (2019) 571(7764):270–4. doi: 10.1038/s41586-019-1324-y PMC7698992 31207604

[80] Khan O Giles JR McDonald S Manne S Ngiow SF Patel KP. Tox transcriptionally and epigenetically programs Cd8(+) T cell exhaustion. Nature (2019) 571(7764):211–8. doi: 10.1038/s41586-019-1325-x PMC6713202 31207603

[81] Mognol GP Spreafico R Wong V Scott-Browne JP Togher S Hoffmann A. Exhaustion-associated regulatory regions in Cd8(+) tumor-infiltrating T cells. Proc Natl Acad Sci USA (2017) 114(13):E2776–E85. doi: 10.1073/pnas.1620498114 PMC5380094 28283662

[82] Wrangle J Wang W Koch A Easwaran H Mohammad HP Vendetti F. Alterations of immune response of non-small cell lung cancer with azacytidine. Oncotarget (2013) 4(11):2067–79. doi: 10.18632/oncotarget.1542 PMC3875770 24162015

[83] Ghoneim HE Fan Y Moustaki A Abdelsamed HA Dash P Dogra P. *De novo* epigenetic programs inhibit pd-1 blockade-mediated T cell rejuvenation. Cell (2017) 170(1):142–57 e19. doi: 10.1016/j.cell.2017.06.007 28648661 PMC5568784

[84] Philip M Fairchild L Sun L Horste EL Camara S Shakiba M. Chromatin states define tumour-specific T cell dysfunction and reprogramming. Nature (2017) 545(7655):452–6. doi: 10.1038/nature22367 PMC5693219 28514453

[85] Ito N Nakamura H Tanaka Y Ohgi S. Lung carcinoma: Analysis of T helper type 1 and 2 cells and T cytotoxic type 1 and 2 cells by intracellular cytokine detection with flow cytometry. Cancer (1999) 85(11):2359–67. doi: 10.1002/(SICI)1097-0142(19990601)85:11<2359::AID-CNCR10>3.0.CO;2-A 10357406

[86] Hu CY Zhang YH Wang T Chen L Gong ZH Wan YS. Interleukin-2 reverses Cd8(+) T cell exhaustion in clinical malignant pleural effusion of lung cancer. Clin Exp Immunol (2016) 186(1):106–14. doi: 10.1111/cei.12845 PMC5011365 27447482

[87] Leem G Jeon M Kim KW Jeong S Choi SJ Lee YJ. Tumour-infiltrating bystander Cd8(+) T cells activated by il-15 contribute to tumour control in non-small cell lung cancer. Thorax (2022) 77(8):769–80. doi: 10.1136/thoraxjnl-2021-217001 34853159

[88] Zhao S Zheng X Zhu X Ning J Zhu K Yan Y. Surgical trauma-induced Ccl2 upregulation mediates lung cancer progression by promoting treg recruitment in mice and patients. Cancer Invest (2022) 40(2):91–102. doi: 10.1080/07357907.2021.1977314 34515610

[89] Eruslanov EB Bhojnagarwala PS Quatromoni JG Stephen TL Ranganathan A Deshpande C. Tumor-associated neutrophils stimulate T cell responses in early-stage human lung cancer. J Clin Invest (2014) 124(12):5466–80. doi: 10.1172/JCI77053 PMC4348966 25384214

[90] Xu F Wei Y Tang Z Liu B Dong J. Tumor−Associated macrophages in lung cancer: Friend or foe? (Review). Mol Med Rep (2020) 22(5):4107–15. doi: 10.3892/mmr.2020.11518 PMC7533506 33000214

[91] Zhang H Liu Z Wen H Guo Y Xu F Zhu Q. Immunosuppressive Trem2(+) macrophages are associated with undesirable prognosis and responses to anti-Pd-1 immunotherapy in non-small cell lung cancer. Cancer Immunol Immunother (2022) 71(10):2511–22. doi: 10.1007/s00262-022-03173-w PMC10991123 35278107

[92] Cai L Liu H Huang F Fujimoto J Girard L Chen J. Cell-autonomous immune gene expression is repressed in pulmonary neuroendocrine cells and small cell lung cancer. Commun Biol (2021) 4(1):314. doi: 10.1038/s42003-021-01842-7 33750914 PMC7943563

[93] Johnson AM Bullock BL Neuwelt AJ Poczobutt JM Kaspar RE Li HY. Cancer cell-intrinsic expression of mhc class ii regulates the immune microenvironment and response to anti-Pd-1 therapy in lung adenocarcinoma. J Immunol (2020) 204(8):2295–307. doi: 10.4049/jimmunol.1900778 PMC7472648 32179637

[94] Wang HM Zhang XH Ye LQ Zhang K Yang NN Geng S. Insufficient Cd100 shedding contributes to suppression of Cd8(+) T-cell activity in non-small cell lung cancer. Immunology (2020) 160(2):209–19. doi: 10.1111/imm.13189 PMC7218665 32149403

[95] Zou JY Su CH Luo HH Lei YY Zeng B Zhu HS. Curcumin converts Foxp3+ regulatory T cells to T helper 1 cells in patients with lung cancer. J Cell Biochem (2018) 119(2):1420–8. doi: 10.1002/jcb.26302 28731226

[96] Zhao Y Shao Q Zhu H Xu H Long W Yu B. Resveratrol ameliorates Lewis lung carcinoma-bearing mice development, decreases granulocytic myeloid-derived suppressor cell accumulation and impairs its suppressive ability. Cancer Sci (2018) 109(9):2677–86. doi: 10.1111/cas.13720 PMC6125446 29959821

[97] Han X Zhao N Zhu W Wang J Liu B Teng Y. Resveratrol attenuates tnbc lung metastasis by down-regulating pd-1 expression on pulmonary T cells and converting macrophages to M1 phenotype in a murine tumor model. Cell Immunol (2021) 368:104423. doi: 10.1016/j.cellimm.2021.104423 34399171

[98] Zheng X Dong L Wang K Zou H Zhao S Wang Y. Mir-21 participates in the pd-1/Pd-L1 pathway-mediated imbalance of Th17/Treg cells in patients after gastric cancer resection. Ann Surg Oncol (2019) 26(3):884–93. doi: 10.1245/s10434-018-07117-6 30565043

[99] Dong L Zheng X Wang K Wang G Zou H. Programmed death 1/Programmed cell death-ligand 1 pathway participates in gastric surgery-induced imbalance of T-helper 17/Regulatory T cells in mice. J Trauma Acute Care Surg (2018) 85(3):549–59. doi: 10.1097/TA.0000000000001903 29554041

[100] Tohme S Yazdani HO Al-Khafaji AB Chidi AP Loughran P Mowen K. Neutrophil extracellular traps promote the development and progression of liver metastases after surgical stress. Cancer Res (2016) 76(6):1367–80. doi: 10.1158/0008-5472.CAN-15-1591 PMC4794393 26759232

[101] Onuma AE Zhang H Gil L Huang H Tsung A. Surgical stress promotes tumor progression: A focus on the impact of the immune response. J Clin Med (2020) 9(12):4096. doi: 10.3390/jcm9124096 33353113 PMC7766515

[102] Lee JW Shahzad MM Lin YG Armaiz-Pena G Mangala LS Han HD. Surgical stress promotes tumor growth in ovarian carcinoma. Clin Cancer Res (2009) 15(8):2695–702. doi: 10.1158/1078-0432.CCR-08-2966 PMC2746852 19351748

[103] Fridlender ZG Sun J Kim S Kapoor V Cheng G Ling L. Polarization of tumor-associated neutrophil phenotype by tgf-beta: “N1” versus “N2” tan. Cancer Cell (2009) 16(3):183–94. doi: 10.1016/j.ccr.2009.06.017 PMC2754404 19732719

[104] Simoncello F Piperno GM Caronni N Amadio R Cappelletto A Canarutto G. Cxcl5-mediated accumulation of mature neutrophils in lung cancer tissues impairs the differentiation program of anticancer Cd8 T cells and limits the efficacy of checkpoint inhibitors. Oncoimmunology (2022) 11(1):2059876. doi: 10.1080/2162402X.2022.2059876 35402081 PMC8993093

[105] Li X Chen Z Ni Y Bian C Huang J Chen L. Tumor-associated macrophages secret exosomal mir-155 and mir-196a-5p to promote metastasis of non-Small-Cell lung cancer. Transl Lung Cancer Res (2021) 10(3):1338–54. doi: 10.21037/tlcr-20-1255 PMC8044469 33889514

[106] Tan B Shi X Zhang J Qin J Zhang N Ren H. Inhibition of rspo-Lgr4 facilitates checkpoint blockade therapy by switching macrophage polarization. Cancer Res (2018) 78(17):4929–42. doi: 10.1158/0008-5472.CAN-18-0152 29967265

[107] DeNardo DG Brennan DJ Rexhepaj E Ruffell B Shiao SL Madden SF. Leukocyte complexity predicts breast cancer survival and functionally regulates response to chemotherapy. Cancer Discov (2011) 1(1):54–67. doi: 10.1158/2159-8274.CD-10-0028 22039576 PMC3203524

[108] Baghdadi M Yoneda A Yamashina T Nagao H Komohara Y Nagai S. Tim-4 glycoprotein-mediated degradation of dying tumor cells by autophagy leads to reduced antigen presentation and increased immune tolerance. Immunity (2013) 39(6):1070–81. doi: 10.1016/j.immuni.2013.09.014 24315994

[109] He Y Rozeboom L Rivard CJ Ellison K Dziadziuszko R Yu H. Mhc class ii expression in lung cancer. Lung Cancer (2017) 112:75–80. doi: 10.1016/j.lungcan.2017.07.030 29191604

[110] Burr ML Sparbier CE Chan KL Chan YC Kersbergen A Lam EYN. An evolutionarily conserved function of polycomb silences the mhc class I antigen presentation pathway and enables immune evasion in cancer. Cancer Cell (2019) 36(4):385–401 e8. doi: 10.1016/j.ccell.2019.08.008 31564637 PMC6876280

[111] Luque MCA Galuppo MK Capelli-Peixoto J Stolf BS. Cd100 effects in macrophages and its roles in atherosclerosis. Front Cardiovasc Med (2018) 5:136. doi: 10.3389/fcvm.2018.00136 30324109 PMC6173139

[112] Basile JR Holmbeck K Bugge TH Gutkind JS. Mt1-mmp controls tumor-induced angiogenesis through the release of semaphorin 4d. J Biol Chem (2007) 282(9):6899–905. doi: 10.1074/jbc.M609570200 17204469

[113] Yang S Wang L Pan W Bayer W Thoens C Heim K. Mmp2/Mmp9-mediated Cd100 shedding is crucial for inducing intrahepatic anti-hbv Cd8 T cell responses and hbv clearance. J Hepatol (2019) 71(4):685–98. doi: 10.1016/j.jhep.2019.05.013 31173811

[114] Dickson RP Erb-Downward JR Martinez FJ Huffnagle GB. The microbiome and the respiratory tract. Annu Rev Physiol (2016) 78:481–504. doi: 10.1146/annurev-physiol-021115-105238 26527186 PMC4751994

[115] Matsuzaki J Tsuji T Luescher I Old LJ Shrikant P Gnjatic S. Nonclassical antigen-processing pathways are required for mhc class ii-restricted direct tumor recognition by ny-Eso-1-Specific Cd4(+) T cells. Cancer Immunol Res (2014) 2(4):341–50. doi: 10.1158/2326-6066.CIR-13-0138 PMC4004114 24764581

[116] Yang M Li Z Tao J Hu H Li Z Zhang Z. Resveratrol induces pd-L1 expression through snail-driven activation of wnt pathway in lung cancer cells. J Cancer Res Clin Oncol (2021) 147(4):1101–13. doi: 10.1007/s00432-021-03510-z PMC7954741 33471184

[117] Li X Wang D Zhao QC Shi T Chen J. Resveratrol inhibited non-small cell lung cancer through inhibiting stat-3 signaling. Am J Med Sci (2016) 352(5):524–30. doi: 10.1016/j.amjms.2016.08.027 27865301

[118] Wright C Iyer AKV Yakisich JS Azad N. Anti-tumorigenic effects of resveratrol in lung cancer cells through modulation of c-flip. Curr Cancer Drug Targets (2017) 17(7):669–80. doi: 10.2174/1568009617666170315162932 PMC6476192 28302032

[119] Li Y Yang Y Liu X Long Y Zheng Y. Prmt5 promotes human lung cancer cell apoptosis *Via* Akt/Gsk3beta signaling induced by resveratrol. Cell Transplant (2019) 28(12):1664–73. doi: 10.1177/0963689719885083 PMC6923546 31665911

[120] Hsieh TC Wu JM. Resveratrol: Biological and pharmaceutical properties as anticancer molecule. Biofactors (2010) 36(5):360–9. doi: 10.1002/biof.105 PMC3655417 20623546

[121] Fan XX Yao XJ Xu SW Wong VK He JX Ding J. (Z)3,4,5,4’-Trans-Tetramethoxystilbene, a new analogue of resveratrol, inhibits gefitinb-resistant non-small cell lung cancer *via* selectively elevating intracellular calcium level. Sci Rep (2015) 5:16348. doi: 10.1038/srep16348 26542098 PMC4635386

[122] van der Windt GJ O’Sullivan D Everts B Huang SC Buck MD Curtis JD. Cd8 memory T cells have a bioenergetic advantage that underlies their rapid recall ability. Proc Natl Acad Sci U.S.A. (2013) 110(35):14336–41. doi: 10.1073/pnas.1221740110 PMC3761631 23940348

[123] Scharping NE Delgoffe GM. Tumor microenvironment metabolism: A new checkpoint for anti-tumor immunity. Vaccines (Basel) (2016) 4(4):46. doi: 10.3390/vaccines4040046 27929420 PMC5192366

[124] Chang CH Qiu J O’Sullivan D Buck MD Noguchi T Curtis JD. Metabolic competition in the tumor microenvironment is a driver of cancer progression. Cell (2015) 162(6):1229–41. doi: 10.1016/j.cell.2015.08.016 PMC4864363 26321679

[125] Frauwirth KA Riley JL Harris MH Parry RV Rathmell JC Plas DR. The Cd28 signaling pathway regulates glucose metabolism. Immunity (2002) 16(6):769–77. doi: 10.1016/s1074-7613(02)00323-0 12121659

[126] Zhao E Maj T Kryczek I Li W Wu K Zhao L. Cancer mediates effector T cell dysfunction by targeting micrornas and Ezh2 *Via* glycolysis restriction. Nat Immunol (2016) 17(1):95–103. doi: 10.1038/ni.3313 26523864 PMC4684796

[127] Ho PC Bihuniak JD Macintyre AN Staron M Liu X Amezquita R. Phosphoenolpyruvate is a metabolic checkpoint of anti-tumor T cell responses. Cell (2015) 162(6):1217–28. doi: 10.1016/j.cell.2015.08.012 PMC4567953 26321681

[128] Munder M Choi BS Rogers M Kropf P. L-arginine deprivation impairs leishmania major-specific T-cell responses. Eur J Immunol (2009) 39(8):2161–72. doi: 10.1002/eji.200839041 PMC2948424 19637195

[129] Altman BJ Stine ZE Dang CV. From Krebs to clinic: Glutamine metabolism to cancer therapy. Nat Rev Cancer (2016) 16(10):619–34. doi: 10.1038/nrc.2016.71 PMC5484415 27492215

[130] Platten M Wick W Van den Eynde BJ. Tryptophan catabolism in cancer: Beyond ido and tryptophan depletion. Cancer Res (2012) 72(21):5435–40. doi: 10.1158/0008-5472.CAN-12-0569 23090118

[131] Julliard W Fechner JH Mezrich JD. The aryl hydrocarbon receptor meets immunology: Friend or foe? a little of both. Front Immunol (2014) 5:458. doi: 10.3389/fimmu.2014.00458 25324842 PMC4183121

[132] Xia H Wang W Crespo J Kryczek I Li W Wei S. Suppression of Fip200 and autophagy by tumor-derived lactate promotes naive T cell apoptosis and affects tumor immunity. Sci Immunol (2017) 2(17):eaan4631. doi: 10.1126/sciimmunol.aan4631 29150439 PMC5774333

[133] Choi SY Collins CC Gout PW Wang Y. Cancer-generated lactic acid: A regulatory, immunosuppressive metabolite? J Pathol (2013) 230(4):350–5. doi: 10.1002/path.4218 PMC3757307 23729358

[134] Pilon-Thomas S Kodumudi KN El-Kenawi AE Russell S Weber AM Luddy K. Neutralization of tumor acidity improves antitumor responses to immunotherapy. Cancer Res (2016) 76(6):1381–90. doi: 10.1158/0008-5472.CAN-15-1743 PMC4829106 26719539

[135] Doedens AL Phan AT Stradner MH Fujimoto JK Nguyen JV Yang E. Hypoxia-inducible factors enhance the effector responses of Cd8(+) T cells to persistent antigen. Nat Immunol (2013) 14(11):1173–82. doi: 10.1038/ni.2714 PMC3977965 24076634

[136] Gropper Y Feferman T Shalit T Salame TM Porat Z Shakhar G. Culturing ctls under hypoxic conditions enhances their cytolysis and improves their anti-tumor function. Cell Rep (2017) 20(11):2547–55. doi: 10.1016/j.celrep.2017.08.071 28903036

[137] Fischer K Hoffmann P Voelkl S Meidenbauer N Ammer J Edinger M. Inhibitory effect of tumor cell-derived lactic acid on human T cells. Blood (2007) 109(9):3812–9. doi: 10.1182/blood-2006-07-035972 17255361

[138] Noman MZ Desantis G Janji B Hasmim M Karray S Dessen P. Pd-L1 is a novel direct target of hif-1alpha, and its blockade under hypoxia enhanced mdsc-mediated T cell activation. J Exp Med (2014) 211(5):781–90. doi: 10.1084/jem.20131916 PMC4010891 24778419

[139] Chen SJ Wang SC Chen YC. The immunotherapy for colorectal cancer, lung cancer and pancreatic cancer. Int J Mol Sci (2021) 22(23):12836. doi: 10.3390/ijms222312836 34884642 PMC8657810

[140] Met O Jensen KM Chamberlain CA Donia M Svane IM. Principles of adoptive T cell therapy in cancer. Semin Immunopathol (2019) 41(1):49–58. doi: 10.1007/s00281-018-0703-z 30187086

[141] Nakajima J Murakawa T Fukami T Goto S Kaneko T Yoshida Y. A phase I study of adoptive immunotherapy for recurrent non-Small-Cell lung cancer patients with autologous gammadelta T cells. Eur J Cardiothorac Surg (2010) 37(5):1191–7. doi: 10.1016/j.ejcts.2009.11.051 20137969

[142] Sakamoto M Nakajima J Murakawa T Fukami T Yoshida Y Murayama T Adoptive immunotherapy for advanced non-small cell lung cancer using zoledronate-expanded gammadelta T cells: A phase I clinical study. J Immunother (2011) 34(2):202–11. doi: 10.1097/CJI.0b013e318207ecfb 21304399

[143] Kakimi K Matsushita H Murakawa T Nakajima J. Gammadelta T cell therapy for the treatment of non-small cell lung cancer. Transl Lung Cancer Res (2014) 3(1):23–33. doi: 10.3978/j.issn.2218-6751.2013.11.01 25806278 PMC4367606

[144] Xu Y Xiang Z Alnaggar M Kouakanou L Li J He J. Allogeneic Vgamma9vdelta2 T-cell immunotherapy exhibits promising clinical safety and prolongs the survival of patients with late-stage lung or liver cancer. Cell Mol Immunol (2021) 18(2):427–39. doi: 10.1038/s41423-020-0515-7 PMC8027668 32939032

[145] Ratto GB Zino P Mirabelli S Minuti P Aquilina R Fantino G. A randomized trial of adoptive immunotherapy with tumor-infiltrating lymphocytes and interleukin-2 versus standard therapy in the postoperative treatment of resected nonsmall cell lung carcinoma. Cancer (1996) 78(2):244–51. doi: 10.1002/(sici)1097-0142(19960715)78:2<244::Aid-cncr9>3.0.Co;2-l 8673999

[146] Melioli G Ratto GB Ponte M Guastella M Semino C Fantino G. Treatment of stage iiib non-Small-Cell lung cancer with surgery followed by infusion of tumor infiltrating lymphocytes and recombinant interleukin-2: A pilot study. J Immunother Emphasis Tumor Immunol (1996) 19(3):224–30. doi: 10.1097/00002371-199605000-00007 8811497

[147] Creelan BC Wang C Teer JK Toloza EM Yao J Kim S. Tumor-infiltrating lymphocyte treatment for anti-Pd-1-Resistant metastatic lung cancer: A phase 1 trial. Nat Med (2021) 27(8):1410–8. doi: 10.1038/s41591-021-01462-y PMC8509078 34385708

[148] Feng K Guo Y Dai H Wang Y Li X Jia H. Chimeric antigen receptor-modified T cells for the immunotherapy of patients with egfr-expressing advanced Relapsed/Refractory non-small cell lung cancer. Sci China Life Sci (2016) 59(5):468–79. doi: 10.1007/s11427-016-5023-8 26968708

[149] Zhang Y Zhang Z Ding Y Fang Y Wang P Chu W. Phase I clinical trial of egfr-specific car-T cells generated by the piggybac transposon system in advanced Relapsed/Refractory non-small cell lung cancer patients. J Cancer Res Clin Oncol (2021) 147(12):3725–34. doi: 10.1007/s00432-021-03613-7 PMC11801842 34032893

[150] Han D Xu Z Zhuang Y Ye Z Qian Q. Current progress in car-T cell therapy for hematological malignancies. J Cancer (2021) 12(2):326–34. doi: 10.7150/jca.48976 PMC7738987 33391429

[151] Springuel L Lonez C Alexandre B Van Cutsem E Machiels JH Van Den Eynde M. Chimeric antigen receptor-T cells for targeting solid tumors: Current challenges and existing strategies. BioDrugs (2019) 33(5):515–37. doi: 10.1007/s40259-019-00368-z PMC6790340 31363930

[152] Xue T Zhao X Zhao K Lu Y Yao J Ji X. Immunotherapy for lung cancer: Focusing on chimeric antigen receptor (Car)-T cell therapy. Curr Probl Cancer (2022) 46(1):100791. doi: 10.1016/j.currproblcancer.2021.100791 34538649

[153] Zeltsman M Dozier J McGee E Ngai D Adusumilli PS. Car T-cell therapy for lung cancer and malignant pleural mesothelioma. Transl Res (2017) 187:1–10. doi: 10.1016/j.trsl.2017.04.004 28502785 PMC5581988

[154] Ke EE Wu YL. Egfr as a pharmacological target in egfr-mutant non-Small-Cell lung cancer: Where do we stand now? Trends Pharmacol Sci (2016) 37(11):887–903. doi: 10.1016/j.tips.2016.09.003 27717507

[155] Sepesi B Cascone T Chun SG Altan M Le X. Emerging therapies in thoracic malignancies-immunotherapy, targeted therapy, and T-cell therapy in non-small cell lung cancer. Surg Oncol Clin N Am (2020) 29(4):555–69. doi: 10.1016/j.soc.2020.06.009 PMC7388816 32883458

[156] He J Xiong X Yang H Li D Liu X Li S. Defined tumor antigen-specific T cells potentiate personalized tcr-T cell therapy and prediction of immunotherapy response. Cell Res (2022) 32(6):530–42. doi: 10.1038/s41422-022-00627-9 PMC9160085 35165422

[157] Horne ZD Jack R Gray ZT Siegfried JM Wilson DO Yousem SA. Increased levels of tumor-infiltrating lymphocytes are associated with improved recurrence-free survival in stage 1a non-Small-Cell lung cancer. J Surg Res (2011) 171(1):1–5. doi: 10.1016/j.jss.2011.03.068 21571304

[158] Kilic A Landreneau RJ Luketich JD Pennathur A Schuchert MJ. Density of tumor-infiltrating lymphocytes correlates with disease recurrence and survival in patients with Large non-Small-Cell lung cancer tumors. J Surg Res (2011) 167(2):207–10. doi: 10.1016/j.jss.2009.08.029 19896677

[159] Ben-Avi R Farhi R Ben-Nun A Gorodner M Greenberg E Markel G. Establishment of adoptive cell therapy with tumor infiltrating lymphocytes for non-small cell lung cancer patients. Cancer Immunol Immunother (2018) 67(8):1221–30. doi: 10.1007/s00262-018-2174-4 PMC11028292 29845338

[160] Bao Y Guo L Mo J. Characterization of gammadelta T cells in patients with non-small cell lung cancer. Oncol Lett (2017) 14(1):1133–40. doi: 10.3892/ol.2017.6191 PMC5494795 28693285

[161] Cheng M Hu S. Lung-resident gammadelta T cells and their roles in lung diseases. Immunology (2017) 151(4):375–84. doi: 10.1111/imm.12764 PMC5506441 28555812

[162] Sebestyen Z Prinz I Dechanet-Merville J Silva-Santos B Kuball J. Translating gammadelta (Gammadelta) T cells and their receptors into cancer cell therapies. Nat Rev Drug Discov (2020) 19(3):169–84. doi: 10.1038/s41573-019-0038-z 31492944

[163] Ghaffari S Khalili N Rezaei N. Crispr/Cas9 revitalizes adoptive T-cell therapy for cancer immunotherapy. J Exp Clin Cancer Res (2021) 40(1):269. doi: 10.1186/s13046-021-02076-5 34446084 PMC8390258

[164] Guan L Han Y Zhu S Lin J. Application of crispr-cas system in gene therapy: Pre-clinical progress in animal model. DNA Repair (Amst) (2016) 46:1–8. doi: 10.1016/j.dnarep.2016.07.004 27519625

[165] Dimitri A Herbst F Fraietta JA. Engineering the next-generation of car T-cells with crispr-Cas9 gene editing. Mol Cancer (2022) 21(1):78. doi: 10.1186/s12943-022-01559-z 35303871 PMC8932053

[166] Lu Y Xue J Deng T Zhou X Yu K Deng L. Safety and feasibility of crispr-edited T cells in patients with refractory non-Small-Cell lung cancer. Nat Med (2020) 26(5):732–40. doi: 10.1038/s41591-020-0840-5 32341578

[167] Guo W Liu S Zhang X Chen Y Qian R Zou Z. The coexpression of multi-immune inhibitory receptors on T lymphocytes in primary non-Small-Cell lung cancer. Drug Des Devel Ther (2017) 11:3367–76. doi: 10.2147/DDDT.S148443 PMC5713689 29238163

[168] Li JX Huang JM Jiang ZB Li RZ Sun A Lai-Han Leung E. Current clinical progress of pd-1/Pd-L1 immunotherapy and potential combination treatment in non-small cell lung cancer. Integr Cancer Ther (2019) 18:1534735419890020. doi: 10.1177/1534735419890020 31838881 PMC7242804

[169] Gadgeel S Rodriguez-Abreu D Speranza G Esteban E Felip E Domine M. Updated analysis from keynote-189: Pembrolizumab or placebo plus pemetrexed and platinum for previously untreated metastatic nonsquamous non-Small-Cell lung cancer. J Clin Oncol (2020) 38(14):1505–17. doi: 10.1200/JCO.19.03136 32150489

[170] Patel SA Weiss J. Advances in the treatment of non-small cell lung cancer: Immunotherapy. Clin Chest Med (2020) 41(2):237–47. doi: 10.1016/j.ccm.2020.02.010 32402359

[171] Postow MA Callahan MK Wolchok JD. Immune checkpoint blockade in cancer therapy. J Clin Oncol (2015) 33(17):1974–82. doi: 10.1200/JCO.2014.59.4358 PMC4980573 25605845

[172] Leach DR Krummel MF Allison JP. Enhancement of antitumor immunity by ctla-4 blockade. Science (1996) 271(5256):1734–6. doi: 10.1126/science.271.5256.1734 8596936

[173] Lynch TJ Bondarenko I Luft A Serwatowski P Barlesi F Chacko R. Ipilimumab in combination with paclitaxel and carboplatin as first-line treatment in stage Iiib/Iv non-Small-Cell lung cancer: Results from a randomized, double-blind, multicenter phase ii study. J Clin Oncol (2012) 30(17):2046–54. doi: 10.1200/JCO.2011.38.4032 22547592

[174] Ellis PM. Anti-angiogenesis in personalized therapy of lung cancer. Adv Exp Med Biol (2016) 893:91–126. doi: 10.1007/978-3-319-24223-1_5 26667340

[175] Rivera LB Meyronet D Hervieu V Frederick MJ Bergsland E Bergers G. Intratumoral myeloid cells regulate responsiveness and resistance to antiangiogenic therapy. Cell Rep (2015) 11(4):577–91. doi: 10.1016/j.celrep.2015.03.055 PMC4438771 25892230

[176] Meder L Schuldt P Thelen M Schmitt A Dietlein F Klein S. Combined vegf and pd-L1 blockade displays synergistic treatment effects in an autochthonous mouse model of small cell lung cancer. Cancer Res (2018) 78(15):4270–81. doi: 10.1158/0008-5472.CAN-17-2176 29776963

[177] Chon HJ Lee WS Yang H Kong SJ Lee NK Moon ES. Tumor microenvironment remodeling by intratumoral oncolytic vaccinia virus enhances the efficacy of immune-checkpoint blockade. Clin Cancer Res (2019) 25(5):1612–23. doi: 10.1158/1078-0432.CCR-18-1932 30538109

[178] Kellish P Shabashvili D Rahman MM Nawab A Guijarro MV Zhang M. Oncolytic virotherapy for small-cell lung cancer induces immune infiltration and prolongs survival. J Clin Invest (2019) 129(6):2279–92. doi: 10.1172/JCI121323 PMC6546459 31033480

[179] Schenk EL Mandrekar SJ Dy GK Aubry MC Tan AD Dakhil SR. A randomized double-blind phase ii study of the Seneca valley virus (Ntx-010) versus placebo for patients with extensive-stage sclc (Es sclc) who were stable or responding after at least four cycles of platinum-based chemotherapy: North central cancer treatment group (Alliance) N0923 study. J Thorac Oncol (2020) 15(1):110–9. doi: 10.1016/j.jtho.2019.09.083 PMC7279615 31605793

[180] de la Rosa G Yang D Tewary P Varadhachary A Oppenheim JJ. Lactoferrin acts as an alarmin to promote the recruitment and activation of apcs and antigen-specific immune responses. J Immunol (2008) 180(10):6868–76. doi: 10.4049/jimmunol.180.10.6868 PMC2408856 18453607

[181] Parikh PM Vaid A Advani SH Digumarti R Madhavan J Nag S. Randomized, double-blind, placebo-controlled phase ii study of single-agent oral talactoferrin in patients with locally advanced or metastatic non-small-cell lung cancer that progressed after chemotherapy. J Clin Oncol (2011) 29(31):4129–36. doi: 10.1200/JCO.2010.34.4127 21969509

[182] Ramalingam S Crawford J Chang A Manegold C Perez-Soler R Douillard JY. Talactoferrin Alfa versus placebo in patients with refractory advanced non-Small-Cell lung cancer (Fortis-m trial). Ann Oncol (2013) 24(11):2875–80. doi: 10.1093/annonc/mdt371 24050956

[183] Lissoni P Tisi E Barni S Ardizzoia A Rovelli F Rescaldani R. Biological and clinical results of a neuroimmunotherapy with interleukin-2 and the pineal hormone melatonin as a first line treatment in advanced non-small cell lung cancer. Br J Cancer (1992) 66(1):155–8. doi: 10.1038/bjc.1992.234 PMC1977913 1322155

[184] Ridolfi L Bertetto O Santo A Naglieri E Lopez M Recchia F. Chemotherapy with or without low-dose interleukin-2 in advanced non-small cell lung cancer: Results from a phase iii randomized multicentric trial. Int J Oncol (2011) 39(4):1011–7. doi: 10.3892/ijo.2011.1099 21720704

