# Corrigendum: T lymphocyte cell: A pivotal player in lung cancer

**DOI:** 10.3389/fimmu.2023.1166352

**Published:** 2023-02-21

**Authors:** Yanan Wu, Meng Yuan, Chenlin Wang, Yanfei Chen, Yan Zhang, Jiandong Zhang

**Affiliations:** ^1^ Department of Oncology, the First Affiliated Hospital of Shandong First Medical University & Shandong Provincial Qianfoshan Hospital, Shandong Key Laboratory of Rheumatic Disease and Translational Medicine, Shandong Lung Cancer Institute, Jinan, China; ^2^ Department of Oncology, Shandong First Medical University & Shandong Academy of Medical Sciences, Jinan, China; ^3^ School of Clinical Medicine, Weifang Medical University, Weifang, China; ^4^ Medical Integration and Practice Center, Cheeloo College of Medicine, Shandong University, Jinan, China

**Keywords:** lung cancer, T lymphocyte, immunotherapy, tumor microenvironment, immunosuppression

In the published article, there was an error in affiliations 1 and 2. Instead of “^1^Department of Oncology, Shandong First Medical University, Jinan, China, ^2^Department of Oncology, the First Affiliated Hospital of Shandong First Medical University & Shandong Provincial Qianfoshan Hospital, Jinan, Shandong, China” it should be “^1^Department of Oncology, the First Affiliated Hospital of Shandong First Medical University & Shandong Provincial Qianfoshan Hospital, Shandong Key Laboratory of Rheumatic Disease and Translational Medicine, Shandong Lung Cancer Institute, Jinan, China, ^2^Department of Oncology, Shandong First Medical University & Shandong Academy of Medical Sciences, Jinan, China”.

In the published article, there was an error in the Funding statement. Instead of the following: “This research was funded by Shandong Natural Science Foundation [ZR2021LSW023 and ZR2022MH111].” The correct Funding statement appears below.

